# Intervening in Symbiotic Cross-Kingdom Biofilm Interactions: a Binding Mechanism-Based Nonmicrobicidal Approach

**DOI:** 10.1128/mBio.00651-21

**Published:** 2021-05-18

**Authors:** H. E. Kim, A. Dhall, Y. Liu, M. Bawazir, H. Koo, G. Hwang

**Affiliations:** a Department of Preventive and Restorative Sciences, School of Dental Medicine, University of Pennsylvania, Philadelphia, Pennsylvania, USA; b Biofilm Research Labs, Levy Center for Oral Health, Department of Orthodontics, Divisions of Pediatric Dentistry and Community Oral Health, School of Dental Medicine, University of Pennsylvania, Philadelphia, Pennsylvania, USA; c Center for Innovation & Precision Dentistry, School of Dental Medicine, and School of Engineering and Applied Sciences, University of Pennsylvania, Philadelphia, Pennsylvania, USA; University of Pittsburgh School of Medicine

**Keywords:** mannan-degrading enzymes, *Streptococcus mutans*, *Candida albicans*, polymicrobial interaction, nonmicrobicidal approach

## Abstract

Early childhood caries is a severe oral disease that results in aggressive tooth decay. Particularly, a synergistic association between a fungus, Candida albicans, and a cariogenic bacterium, Streptococcus mutans, promotes the development of hard-to-remove and highly acidic biofilms, exacerbating the virulent damage. These interactions are largely mediated via glucosyltransferases (GtfB) binding to mannans on the cell wall of C. albicans. Here, we present an enzymatic approach to target GtfB-mannan interactions in this cross-kingdom consortium using mannan-degrading exo- and endo-enzymes. These exo- and endo-enzymes are highly effective in reducing biofilm biomass without killing microorganisms, as well as alleviating the production of an acidic pH environment conducive to tooth decay. To corroborate these results, we present biophysical evidence using single-molecule atomic force microscopy, biofilm shearing, and enamel surface topography analyses. Data show a drastic decrease in binding forces of GtfB to C. albicans (∼15-fold reduction) following enzyme treatment. Furthermore, enzymatic activity disrupted biofilm mechanical stability and significantly reduced human tooth enamel demineralization without cytotoxic effects on gingival keratinocytes. Our results represent significant progress toward a novel nonbiocidal therapeutic intervention against pathogenic bacterial-fungal biofilms by targeting the interkingdom receptor-ligand binding interactions.

## INTRODUCTION

Early childhood caries (ECC), an aggressive form of tooth decay with rampant caries lesions (1, 2), is associated with frequent consumption of fermentable carbohydrates and poor oral hygiene (3, 4). The microorganisms predominantly identified in ECC belong to *Streptococcus* spp., *Candida* spp., *Lactobacillus* spp., *Actinomyces* spp., and *Veillonella* spp. (5–9). Particularly, Candida albicans, an opportunistic fungal pathogen, is known to interact with cariogenic Streptococcus mutans to form biofilms associated with ECC (3, 10). Symbiotic and synergistic interactions between these two kingdoms reinforce biofilm pathogenesis and the virulence of ECC (11, 12).

Given the aggressive damage caused by ECC (1, 2, 6) and its characterization as a polymicrobial disease with cross-kingdom consortia that develop hard-to-remove and highly acidic biofilms, there is a great need to strategically develop a targeted measure to effectively prevent cross-kingdom interactions and subsequent biofilm development. While there have been endeavors to treat fungal-involved biofilm-associated diseases by use of antibacterial or antifungal agents (13–15), these often exhibit limited efficacy due to a lack of targeting polymicrobial interactions. Furthermore, it is worth noting that these antimicrobials may disrupt ecological microbiota and/or induce drug resistance over time, providing significant limitations for preventive measures with long-term use.

The cross-kingdom adhesion between S. mutans and C. albicans is dependent on the availability of sucrose and secreted bacterial exoenzymes (e.g., glucosyltransferases [Gtfs]) (16, 17). Secreted Gtfs use sucrose to produce extracellular polymeric substances (EPS), in particular insoluble polysaccharides, which in turn form the extracellular matrix in cariogenic biofilms (18). Specifically, we have previously shown that GtfB from S. mutans strongly binds to the C. albicans cell wall and it leads to the enhanced production of EPS (19). Such elevated EPS amounts, in turn, lead to an increased number of binding sites for S. mutans (17, 20), which promotes coadhesion and subsequent biofilm formation *in vivo* (11, 12). Furthermore, we have previously elucidated the mechanism of this biochemical interaction between GtfB and C. albicans; mannans on the C. albicans surface act as receptors for GtfB, thereby mediating the cross-kingdom interaction (11). We have also shown that *N*- and *O*-linked mannan-defective mutant strains exhibit severely reduced GtfB binding relative to wild-type strains, resulting in impaired maturation of cross-kingdom biofilms with S. mutans (11). These findings provide an opportunity to develop novel approaches specifically targeting the adhesive interaction between S. mutans and C. albicans without necessarily being toxic to surrounding microbiota and tissues in the oral cavity.

Bolstered by our previous identification of the interkingdom receptor-ligand binding interaction, we hypothesized that mannan-degrading enzymes (MDEs) can disrupt S. mutans*-*C. albicans interactions by reducing the number of binding sites available to form a mature cross-kingdom biofilm. Here, we employed three MDEs (the endoenzyme 1,4-β-mannanase and the two exoenzymes α- and β-mannosidase) to disrupt S. mutans*-*C. albicans biofilm interactions as a targeted strategy to prevent ECC. We comprehensively assessed the activity of MDEs in various buffers and human saliva. We also quantified the ability of MDEs to degrade mannans on the C. albicans cell wall and to reduce the binding potential with GtfB. Then, we determined the efficacy of MDEs to target S. mutans*-*C. albicans biofilms cultured on hydroxyapatite discs in human saliva to mimic physiological conditions in the oral cavity. We found that β-mannanase significantly diminished the cross-kingdom biofilm development, resulting in an ∼2.5-fold reduction of total biomass compared with the vehicle control. In addition, the mechanical stability of biofilms was remarkably weakened by β-mannanase treatment, causing nearly complete surface detachment when exposed to mild shear stress. Notably, the acidic environment induced by the cross-kingdom biofilms was alleviated, showing an elevated pH during biofilm development and reduced demineralization of the tooth enamel surface. To corroborate these results, we utilized single-molecule atomic force microscopy (AFM) to measure GtfB-C. albicans binding forces. Data revealed a significant reduction in average binding forces for MDE-treated C. albicans (up to ∼15-fold reduction versus vehicle control). Finally, we confirmed that MDEs were devoid of microbiocidal activity while showing no cytotoxicity against human gingival keratinocytes. Such a nontoxic but highly specific targeting of the interkingdom receptor-ligand binding interactions may lead to precision therapies for preventing biofilms associated with severe childhood dental caries.

## RESULTS

### Enzyme activity in MES buffer and saliva.

MDEs were chosen to degrade mannans on the cell wall of C. albicans and thereby reduce the incidence of the S. mutans*-*C. albicans biofilm interactions. As the efficacies of MDEs in cleaving mannans could be varied depending on their site of action, we tested both exo- (α-mannosidase and β-mannosidase) and endo- (β-mannanase) mannan-degrading enzymes, where the exoenzymes would hydrolyze terminal mannose residues while the endoenzyme would randomly hydrolyze mannosidic linkages within mannans. Before demonstrating their use, we confirmed that the MDEs were active against their respective substrates in our experimental conditions and optimized the treatment time. The recommended buffers for α-mannosidase, β-mannosidase, and β-mannanase are 2-(*N*-morpholino) ethanesulfonic acid hydrate (MES), sodium maleate, and phosphate buffer, respectively. To ensure consistency during experiments and to reproducibly compare results, we compared the activities of MDEs in a single buffer (100 mM MES buffer with 2.5 mM CaCl_2_ at pH 6.5 at 37°C). As shown in Fig. S1 in the supplemental material, α-mannosidase showed 1.07-fold, β-mannosidase showed 1.07-fold, and β-mannanase showed 0.93-fold enzyme activity in the MES buffer (versus reported values from the manufacturers). This indicates that all the MDEs tested in this study exhibited similar levels of enzyme activities when they were suspended in the MES buffer. Therefore, we measured all the enzyme activities at 5, 10, 30, and 60 min in MES buffer to determine optimal condition.

10.1128/mBio.00651-21.1 FIG S1 Enzyme activity in MES versus recommended buffer. Activities were compared for (A) α-mannosidase, (B) β-mannosidase, and (C) β-mannanase. All MDEs displayed similar activity in the MES buffer compared to the recommended buffer by the manufacturer (*n* ≥ 3). Download FIG S1, DOCX file, 0.1 MB. Copyright © 2021 Kim et al. 2021 Kim et al. https://creativecommons.org/licenses/by/4.0/ This content is distributed under the terms of the Creative Commons Attribution 4.0 International license.

**FIG 1:**
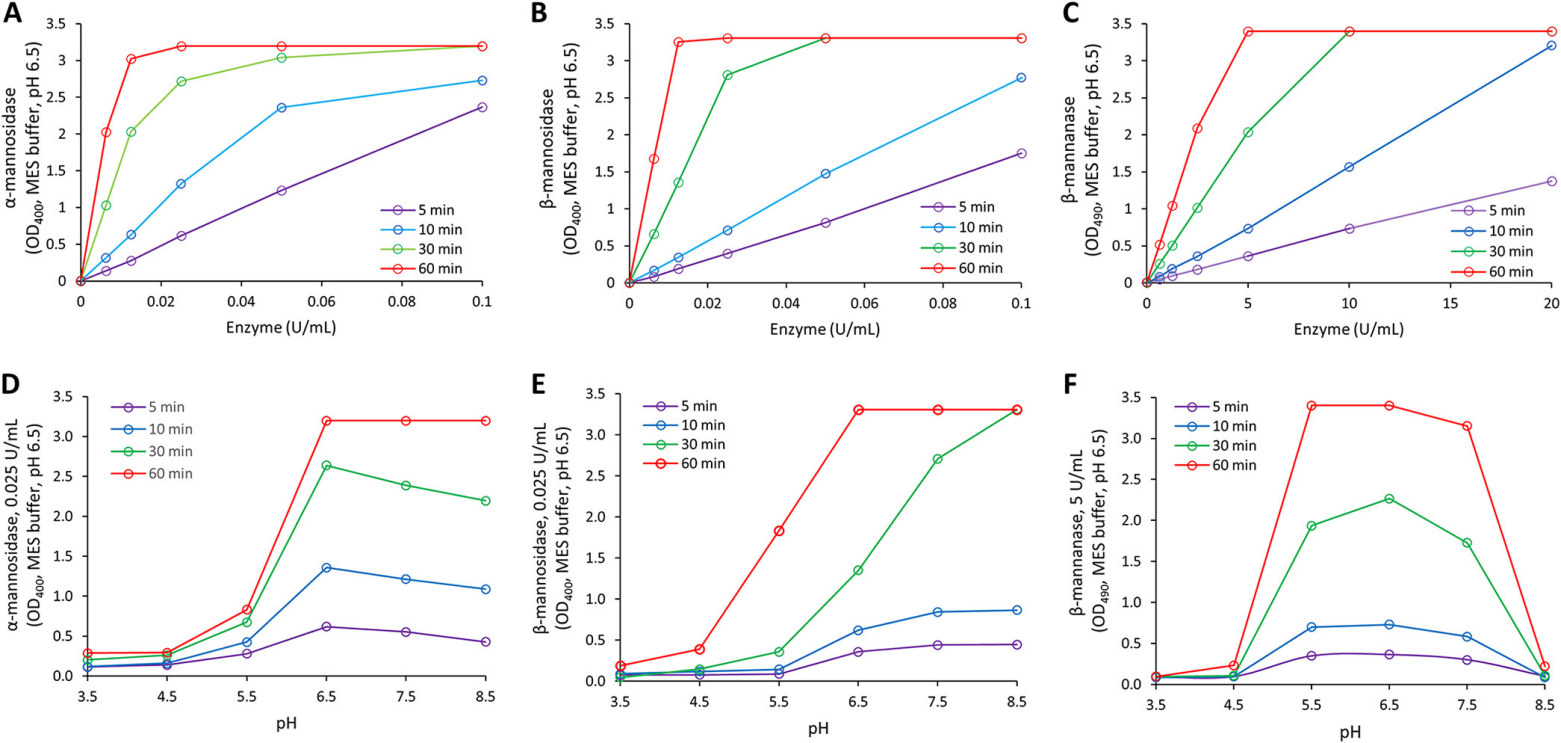
Activity profiles for MDEs in MES buffer. Enzyme activities were measured at different time points for α-mannosidase (A), β-mannosidase (B), and β-mannanase (C). All MDEs had similar activity profiles for all time points. Activities of enzymes in acidic to neutral pH ranges were also determined for α-mannosidase (D), β-mannosidase (E), and β-mannanase (F). (*n* ≥ 3).

The activity profiles at different time points and pH values are depicted in Fig. 1. As shown, similar activity profiles were observed for all the tested conditions. The activities were saturated at higher units and there was discernible activity at as early as 5 min for all MDEs (Fig. 1A to C). The pH profiles for α-mannosidase and β-mannosidase were similar; the highest activity was observed at and near pH 6.5 (Fig. 1D and E). For β-mannanase, the pH profile peaked near pH 6.5 but had a much sharper dip beyond pH 7.0 (Fig. 1F). Since the antibiofilm assays were conducted in human saliva, we also measured the activity profiles with membrane-filtered saliva as the buffering system instead of MES buffer (Fig. S2). Results indicated similar profiles as Fig. 1 for all MDEs.

10.1128/mBio.00651-21.2 FIG S2 Activity profiles for MDEs in saliva. Activities were measured at different time points for (A) α-mannosidase, (B) β-mannosidase, and (C) β-mannanase. All MDEs had similar activity profiles for all time points. pH profiles were measured for (D) α-mannosidase, (E) β-mannosidase, and (F) β-mannanase. All the profiles in saliva were similar to the profiles in the MES buffer (*n* ≥ 3). Download FIG S2, DOCX file, 0.2 MB. Copyright © 2021 Kim et al. 2021 Kim et al. https://creativecommons.org/licenses/by/4.0/ This content is distributed under the terms of the Creative Commons Attribution 4.0 International license.

### Degradation of C. albicans cell wall and GtfB binding potential.

**FIG 2:**
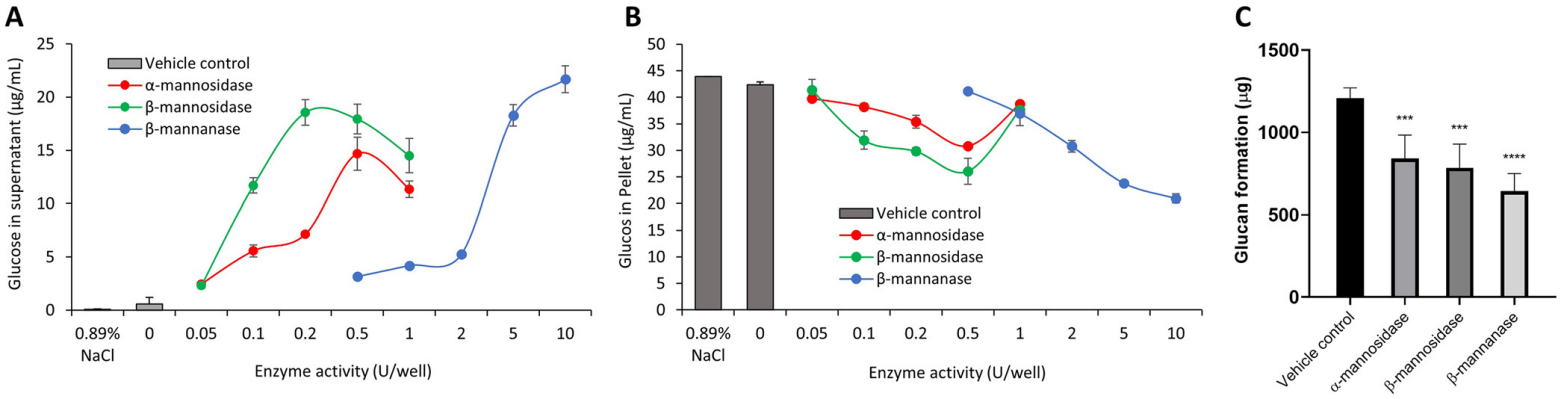
Effect of MDEs on the cell wall of C. albicans and its binding potential with GtfB. Dose-dependent degradation of the cell wall mannans in the supernatant (A) and a corresponding decrease in the pellet of C. albicans (B), and the amount of glucans formed on each C. albicans with or without MDE treatment (C). The amount of mannans on MDE-treated C. albicans in supernatant increased while it decreased from the microbial pellet. In the presence of sucrose, smaller amounts of bound GtfB in MDE-treated C. albicans led to reduced glucan formation. Panel C statistics employed one-way ANOVA with *P < *0.0001 *post hoc*; ***, *P < *0.001; **** *P < *0.0001 against vehicle control using Dunnett’s method (*n* ≥ 3).

Since the MDEs displayed activity against their respective substrates within 5 min and, with our overall aim being to develop a feasible therapeutic intervention strategy to limit biofilm interactions in ECC, we chose the optimal treatment time as 5 min for all our experiments. After selecting an optimal treatment time, we demonstrated enzymatic cell wall degradation of C. albicans by calculating the glucose concentration in the supernatant and pellet (μg/mL) after treatment. We observed a dose-dependent increase in supernatant glucose concentration with increasing enzyme units for all MDEs (Fig. 2A). Consequently, there was a similar decrease in glucose concentration in the pellet, indicating reduced mannan components on MDE-treated C. albicans (Fig. 2B). From these results, we determined the optimal enzyme units for cell wall mannan degradation as 0.5, 0.2, and 10 U/well for α-mannosidase, β-mannosidase, and β-mannanase, respectively.

We have previously shown that mannans on the cell wall of C. albicans mediate GtfB binding to modulate S. mutans*-*C. albicans biofilm development (11). To demonstrate the effect of cell wall degradation via MDEs on the binding of GtfB to C. albicans, we determined the binding potential for GtfB on the surface of C. albicans. Each group of C. albicans with or without enzyme treatment was incubated with equal amounts of GtfB and sucrose to compare the amount of glucans formed on C. albicans. C. albicans treated with optimal units of MDEs for 5 min showed decreased glucan formation compared to the vehicle control (Fig. 2C). Overall, β-mannanase was most effective (∼50% decrease in glucan formation), followed by β-mannosidase (∼35% decrease) and α-mannosidase (∼30% decrease). Our data indicate that MDEs degraded the cell wall of C. albicans and this led to fewer sites available for the binding of GtfB. Subsequently, in the presence of sucrose, this led to smaller amounts of glucans formed.

### Disruption of S. mutans-C. albicans biofilm development.

**FIG 3:**
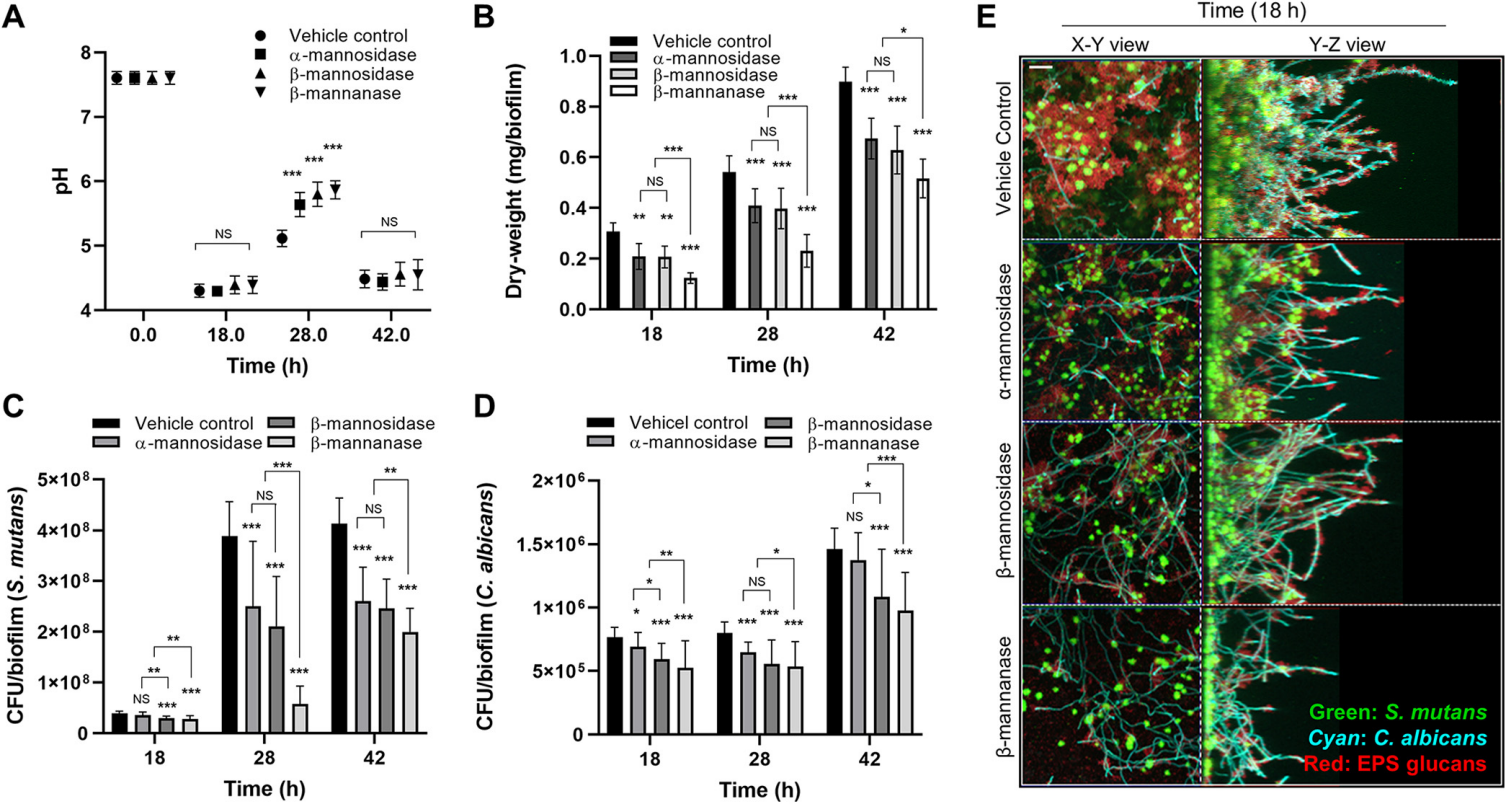
Efficacy of MDEs against S. mutans*-*C. albicans biofilms. (A) The pH of biofilm supernatant. (B) Dry weight per biofilm. (C) CFU of S. mutans per biofilm. (D) CFU of C. albicans per biofilm. At optimal units, all MDEs had a significant antibiofilm effect on S. mutans*-*C. albicans biofilms as measured at 18, 28, and 42 h. (E) Representative confocal images of untreated and MDE-treated biofilms at 18 h. Scale bar indicates 20 μm. Statistics: *** represents *P < *0.001 for unpaired *t* tests against the vehicle control (*n* ≥ 3).

Next, we tested the efficacy of the antibiofilm activity of MDEs using a well-established biofilm assay on hydroxyapatite discs (21). Biofilms were cultured in human saliva to more closely mimic the physiological condition as depicted in Fig. S3. To assess the efficacy of a predetermined dose of MDEs (0.5, 0.2, or 10 U/well of α-mannosidase, β-mannosidase, or β-mannanase, respectively) on the cross-kingdom biofilm disruption, we treated biofilms following the regimen and comprehensively analyzed biofilm properties by measuring the pH of biofilm supernatant, dry weight, and CFU of biofilms (Fig. 3).

10.1128/mBio.00651-21.3 FIG S3 Procedure to measure the antibiofilm activity of MDEs. C. albicans were pretreated with MDEs for 5 min before seeding. MDE treatments were then carried out at 6, 18, and 28 h. Download FIG S3, DOCX file, 0.2 MB. Copyright © 2021 Kim et al. 2021 Kim et al. https://creativecommons.org/licenses/by/4.0/ This content is distributed under the terms of the Creative Commons Attribution 4.0 International license.

Salivary pH values under 5.5 are critical for tooth demineralization (22). For the vehicle control, pH values remained below 5.5, implying an acidic microenvironment conducive to tooth demineralization. At 28 h, in comparison to the vehicle control pH value of 5.08, the pH values rose close to pH 6 when treated with MDEs (Fig. 3A). This is critical, as all three MDEs elevated the pH beyond the critical value of 5.5, signifying an alleviation of the acidic microenvironment.

We also measured the dry weight of biofilms (Fig. 3B). Encouragingly, there were significant reductions in the dry weights for all biofilms treated with MDEs in comparison to the vehicle control. This trend was observed at all time points (18, 28, and 42 h). Overall, the MDEs led to a maximum reduction of dry weight at 28 h. The fold reductions in comparison to the vehicle control were 2.5 for β-mannanase and 1.4 for α- and β-mannosidase. This trend was also observed in the drops in CFU/biofilm (Fig. 3C and D). The drops were greater for S. mutans than C. albicans. This suggests that the loss of binding sites for GtfB on the cell walls of C. albicans prevented S. mutans from dense networking with C. albicans.

To further understand the differences in biofilm properties between samples treated with MDEs and the vehicle control, we investigated the microbial growth and tertiary structures of the biofilms using confocal microscopy (Fig. 3E). Representative confocal images for 18-h biofilms depict a drastic drop in the amount of produced EPS, S. mutans-C. albicans mutualization, and biofilm thickness. Clearly, β-mannanase was most effective, followed by β-mannosidase and α-mannosidase. We confirmed this result with quantitative determinations of biovolume (μm^3^/μm^2^) for each channel (S. mutans, C. albicans, and EPS; Fig. S4).

10.1128/mBio.00651-21.4 FIG S4 Quantification of biovolumes for S. mutans, C. albicans, and EPS with MDEs treatment. Biovolumes (μm^3^/μm^2^) from confocal images for (A) S. mutans, (B) C. albicans, (C) EPS, and (D) total. All MDEs led to a reduction in biovolume of S. mutans, C. albicans, and EPS. Statistics: one-way ANOVA with *P < *0.0001 *post hoc*; *, *P < *0.05; **, *P < *0.01; ***, *P < *0.001; ****, *P < *0.0001 against vehicle control using Dunnett’s method (*n* ≥ 3). Download FIG S4, DOCX file, 0.2 MB. Copyright © 2021 Kim et al. 2021 Kim et al. https://creativecommons.org/licenses/by/4.0/ This content is distributed under the terms of the Creative Commons Attribution 4.0 International license.

### Effect of MDE treatment on the mechanical stability of biofilms.

**FIG 4:**
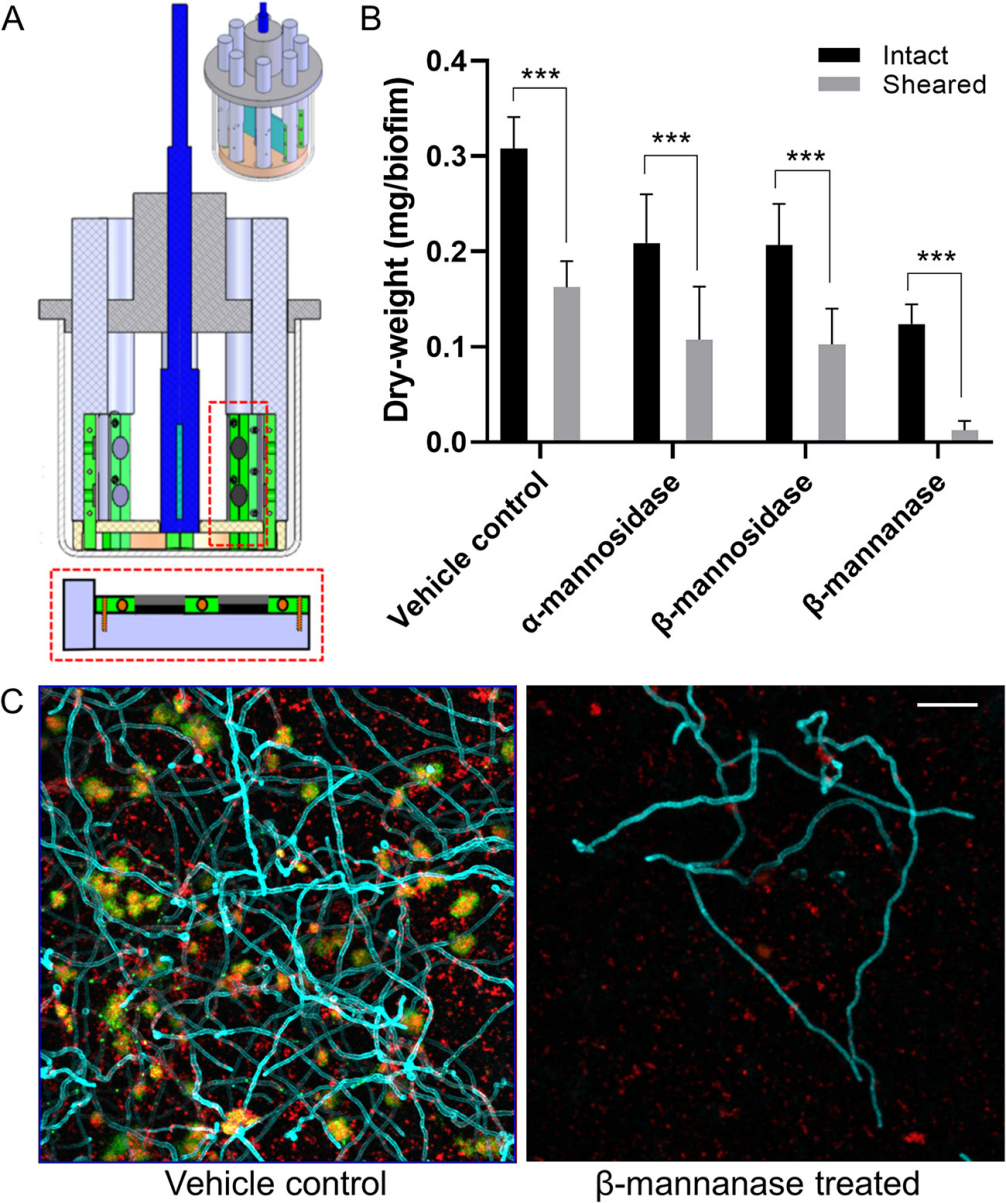
Effect of MDE treatment on the mechanical stability of S. mutans*-*C. albicans biofilms. (A) Schematic diagram of shear-induced biofilm mechanical strength tester. (B) Remaining biofilm biomass before and after applying shear stress of 0.18 N/m^2^ for 10 min. (C) Representative confocal images of biofilms after shearing. Scale bar indicates 20 μm. Statistics: *** represents *P < *0.001 for unpaired *t* tests against the intact biofilms (*n* ≥ 3).

Disruption of S. mutans*-*C. albicans synergistic interactions may weaken the mechanical stability of biofilms. As shown, the amount of biomass and EPS were markedly altered (Fig. 3E) when biofilms were treated with MDEs. Particularly, we observed large clumps detached from biofilms after β-mannanase treatment (data not shown). Thus, we investigated whether our enzymatic strategy could facilitate biofilm removal using a custom-built device (Fig. 4A) that produces shear forces to detach biofilms from the saliva-coated hydroxyapatite (sHA) surfaces (11, 23).

We determined the ability of 18-h biofilms to withstand mechanical removal under shear stress by measuring the amount of biofilm that remained on the sHA before and after applying an estimated shear force (0.18 N/m^2^). Our data showed that MDE-treated biofilms were more susceptible to surface detachment by shear force than vehicle control biofilms (Fig. 4B). This effect was more pronounced following β-mannanase treatment, showing almost complete biofilm removal (∼90% versus unsheared). Furthermore, representative confocal images of sheared biofilms clearly showed that most of the S. mutans microcolonies and C. albicans were detached from the disc surface when treated with β-mannanase, while untreated biofilms still contained numerous sizeable microcolonies and hyphal forms of C. albicans across the surface despite applied shear force (Fig. 4C).

### Effect of MDE treatment on the enamel surface demineralization.

**FIG 5:**
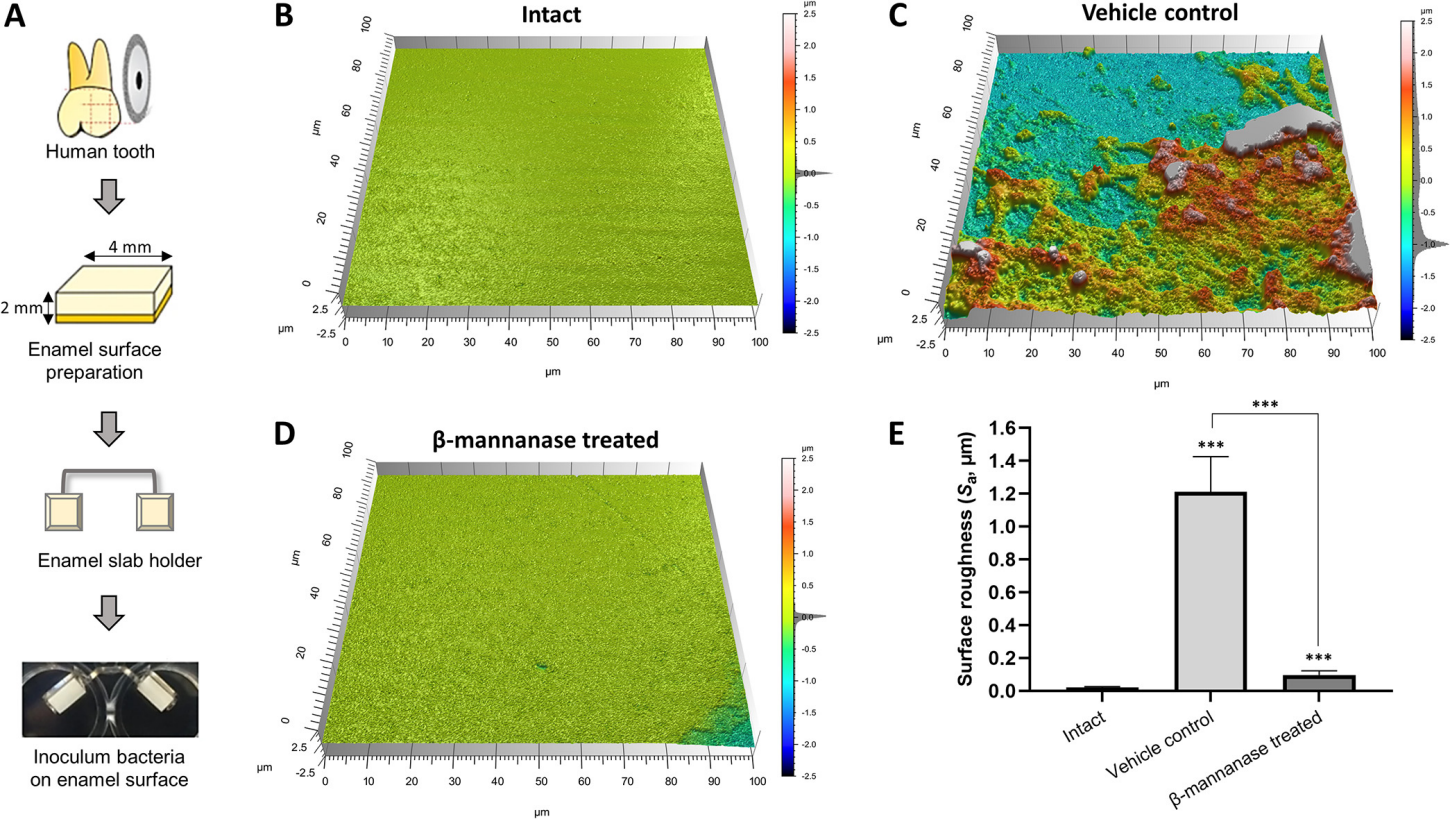
Demineralization of human enamel surface by S. mutans*-*C. albicans biofilms with or without β-mannanase treatment. (A) Schematic diagram for human enamel slab preparation. Shown are representative confocal surface-topography of intact enamel (B), enamel after forming biofilms without β-mannanase treatment (C), and enamel after forming biofilms with β-mannanase treatment (D). Panels (C) and (D) were scanned after removing biofilms from the enamel. (E) Average surface roughness values of intact enamel slabs and slabs that had biofilms with or without β-mannanase treatment; ***, *P < *0.001.

Reduced biofilm biomass and elevated pH by MDE treatment (Fig. 3) may also reduce tooth demineralization. Therefore, we investigated the level of enamel demineralization by culturing S. mutans*-*C. albicans biofilms (with or without β-mannanase treatment) on the human enamel slab (Fig. 5A) in saliva supplemented with 1% sucrose. By culturing biofilms for 5 days on human enamel slabs, we observed similar patterns of pH, biofilm biomass, and CFU to that we observed with the HA disc model (Fig. S5). Then, we inspected the impact on enamel surface integrity by the treated biofilms both visually and quantitatively using confocal surface topographical analysis (24, 25). A smooth and flat surface was observed from the intact surface prior to biofilm formation (Fig. 5B). However, the enamel surfaces underneath untreated S. mutans*-*C. albicans biofilms showed significantly eroded surfaces (Fig. 5C). In marked contrast, mostly intact enamel surface was observed when S. mutans*-*C. albicans biofilms were treated with β-mannanase (Fig. 5D). This observation was supported by quantitative analysis of arithmetical mean height (*S_a_*) following ISO 25178 (26). Overall, enamel surfaces eroded by untreated S. mutans*-*C. albicans biofilms exhibited ∼13-fold higher *S_a_* than those from β-mannanase treated S. mutans*-*C. albicans biofilms (Fig. 5E).

10.1128/mBio.00651-21.5 FIG S5 Efficacy of MDEs against S. mutans*-*C. albicans biofilms on human enamel slab. (A) the pH of biofilm supernatant, (B) dry weight per biofilm, (C) CFU of S. mutans and C. albicans per biofilm. Statistics: ** represents *P < *0.01 for unpaired *t* tests against the vehicle control (*n* ≥ 3). Download FIG S5, DOCX file, 0.1 MB. Copyright © 2021 Kim et al. 2021 Kim et al. https://creativecommons.org/licenses/by/4.0/ This content is distributed under the terms of the Creative Commons Attribution 4.0 International license.

### GtfB-C. albicans cell wall adhesion force for mannan-degraded C. albicans.

**FIG 6:**
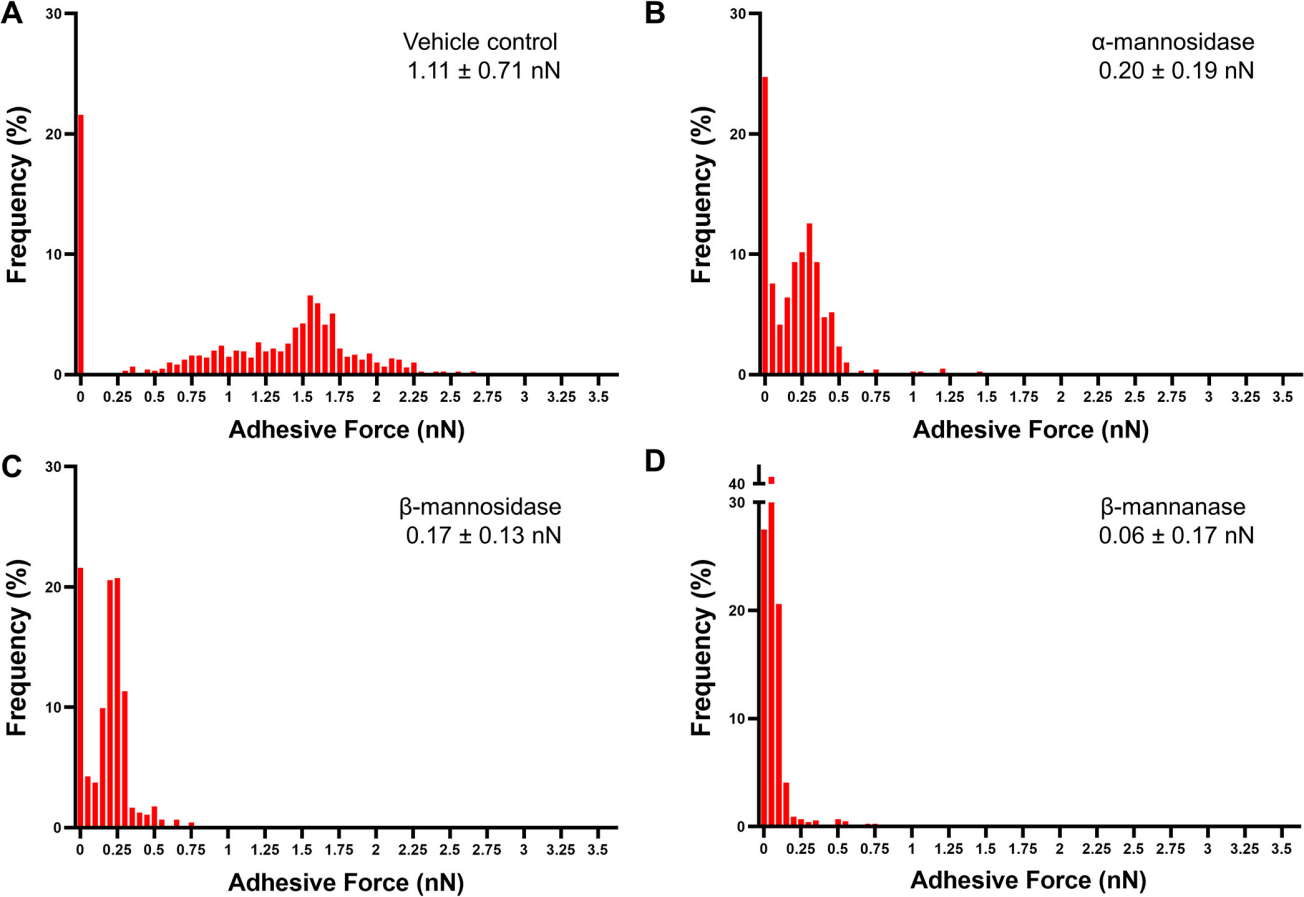
Binding forces of GtfB to MDE treated C. albicans. Shown are adhesion force histograms of GtfB to untreated C. albicans (A), α-mannosidase treated C. albicans (B), β-mannosidase treated C. albicans (C), and β-mannanase treated C. albicans (D) at optimal units and 5 min of treatment. All MDEs drastically reduced the binding forces between GtfB and the cell wall of C. albicans. Kolmogorov-Smirnov tests were used to compare frequency distributions of untreated versus each MDE (*P* < 0.0001 in each case) (*n* ≥ 3).

We have previously shown that GtfB binding strength to the surface of mannan-defective C. albicans was significantly reduced, which resulted in attenuated cross-kingdom biofilm development and tooth demineralization *in vivo* (11). In this study, we observed that the use of MDEs could degrade the cell wall of C. albicans and thus limit biofilm interactions (Fig. 2 and 3). Thus, we corroborated the proposed mechanisms of S. mutans-C. albicans interaction via biophysical measurements of GtfB-C. albicans binding forces for MDE-treated C. albicans using single-molecule AFM. We observed a dose-dependent reduction in GtfB-C. albicans binding forces (Fig. 6) following a similar pattern to that found in mannan-defective strains of C. albicans (11). Untreated C. albicans demonstrated strong binding forces of 1 to 2 nN toward GtfB (Fig. 6A). These forces were significantly reduced when C. albicans was treated with MDEs at optimal units for 5 min; we observed a drastic shift of GtfB binding distribution toward zero adhesive force (Fig. 6B to D). These shifts significantly reduced the average binding forces of GtfB to the surface of α-mannosidase- or β-mannosidase-treated C. albicans up to 5-fold (∼0.2 nN; Fig. 6B and C). Particularly, GtfB binding failure was almost doubled when C. albicans was treated with the endoenzyme β-mannanase, resulting in close to zero average binding force (0.06 nN, an ∼15-fold reduction versus vehicle control; Fig. 6D). These data confirm the trend found in bioassays to measure cell wall degradation and GtfB binding potential of MDE-treated C. albicans (Fig. 2), as well as the antibiofilm effect against cross-kingdom biofilms (Fig. 3).

### Cytotoxicity of MDEs against human gingival keratinocytes.

**FIG 7:**
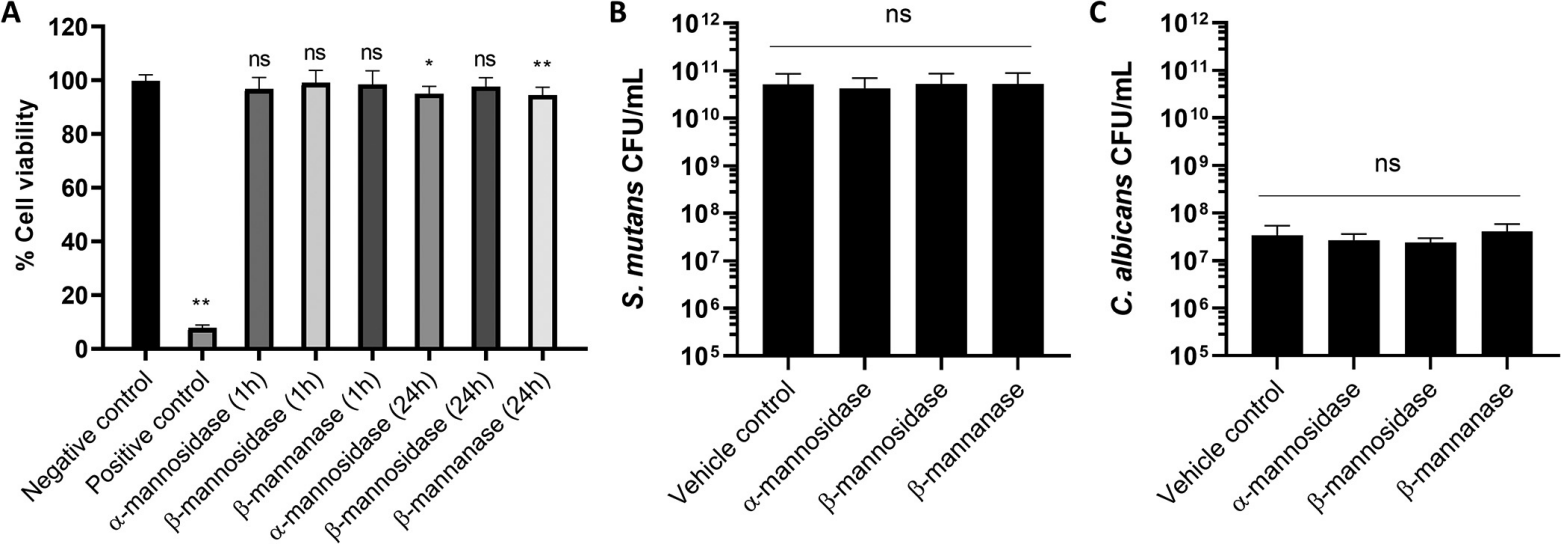
Toxicity assay of MDEs on microbes and human gingival keratinocytes. (A) Normalized cell viability for HGKs after exposure to optimal units of MDEs for 1 h and 24 h, and CFU values for S. mutans (B) and C. albicans (C) after treatment with optimal units of MDEs for 5 min. No loss in HGK cell viability was observed for MDE treatments. Negative control and positive control represent vehicle control and 3% H_2_O_2_ control, respectively. There were no significant differences in microbial cell viability with or without MDE treatment. Statistics: one-way ANOVA with *P < *0.01; *post hoc*: ** represents *P < *0.0001 against vehicle control using Dunnett’s method (*n* ≥ 3); ns, not significant.

For the proposed enzymatic treatment strategy to be sustainable in biofilm disruption therapy, it should not cause antimicrobial resistance nor be toxic toward adjacent human cells in the oral cavity. Therefore, we evaluated the microbicidal effect and cytotoxicity of our MDEs. As expected, none of the MDEs tested in this study exhibited meaningful microbicidal effect; MDEs altered the growth kinetics of neither S. mutans nor C. albicans (Fig. S6). Similarly, there was no discernible drop in CFU/mL for either S. mutans or C. albicans when they were exposed to different MDE units, including optimal units (Fig. 7B and C, and Fig. S7). We performed MTT assays on human gingival keratinocytes to depict the loss in % cell viability after exposure to MDEs at optimal units for 1 h and 24 h. We included the vehicle group as a negative control and a 3% H_2_O_2_-treated group as a positive control (where the keratinocytes would not survive). The keratinocytes displayed no significant drop in cell viability (all > 90%) when treated with any MDEs for either 1 h or 24 h exposure (Fig. 7A). Collectively, our nonmicrobicidal tactic targeting the receptor-ligand binding domain for cross-kingdom interactions using MDEs exhibited great potency in suppressing S. mutans-C. albicans biofilm interactions by degrading the mannans on C. albicans cell wall, without displaying microbiocidal effects or harming human gingival keratinocytes.

10.1128/mBio.00651-21.6 FIG S6 Growth kinetics of S. mutans and C. albicans after treatment with MDEs. Growth curves for C. albicans after treatment with (A) α-mannosidase, (B) β-mannosidase, and (C) β-mannanase. Growth curves for S. mutans after treatment with (D) α-mannosidase, (E) β-mannosidase, and (F) β-mannanase. All MDEs did not affect the growth curves of both microorganisms (*n* ≥ 3). Download FIG S6, DOCX file, 0.2 MB. Copyright © 2021 Kim et al. 2021 Kim et al. https://creativecommons.org/licenses/by/4.0/ This content is distributed under the terms of the Creative Commons Attribution 4.0 International license.

10.1128/mBio.00651-21.7 FIG S7 Microbicidal activity of MDEs at different units. CFU/mL for both S. mutans and C. albicans remained unchanged with the addition of (A) α-mannosidase, (B) β-mannosidase, and (C) β-mannanase. MDEs did not lead to a reduction in CFU/mL for either microbe (*n* ≥ 3). Download FIG S7, DOCX file, 0.2 MB. Copyright © 2021 Kim et al. 2021 Kim et al. https://creativecommons.org/licenses/by/4.0/ This content is distributed under the terms of the Creative Commons Attribution 4.0 International license.

## DISCUSSION

Given its prevalence across all demographic and social variables, ECC poses a serious public health issue in both developing and industrialized countries (27). Among the various factors affecting ECC development, heavy infection by S. mutans and C. albicans under a sugar-rich diet has been shown to be an important microbiological feature in severe ECC (3, 6, 28). Our previous biophysical and *in vivo* studies using mannosylation-defective C. albicans unveiled the governing mechanism of S. mutans*-*C. albicans biofilms interactions (11). Furthermore, it highlighted the possibility of a highly precise strategy to disrupt this pathogenic bacterial-fungal interaction by selectively targeting the GtfB-to-mannans binding mechanism on the C. albicans cell wall surface. While binding domain targeting has been widely applied to interfere with various viral infections, such a strategy remains underexplored for preventing synergistic cross-kingdom biofilm interactions.

Inspired by previous findings, we investigated an enzymatic approach that can specifically degrade mannans on the C. albicans cell wall to interrupt S. mutans*-*C. albicans biofilm interactions using biophysical, biochemical, and microbiological methods. MDEs effectively degraded mannans on C. albicans (Fig. 2A and B), disrupted GtfB binding to C. albicans (Fig. 2C), and attenuated S. mutans*-*C. albicans biofilm development and acidogenicity (Fig. 3). Dose-dependent degradation of the C. albicans cell wall was accompanied by an increased reduction of GtfB binding and subsequent disruption of localized glucan production. Overall, β-mannanase was significantly (up to 2.5-fold) more effective than β-mannosidase and α-mannosidase in exerting antibiofilm activity. This included a significant reduction in total biofilm biomass as well as the content of individual biofilm components (Fig. 3 and Fig. S4). Furthermore, biofilms treated with β-mannanase inflicted minimal tooth enamel surface demineralization (Fig. 5).

The antibiofilm mechanism of this approach was further assessed using biophysical methods. In our previous study, we observed that GtfB-C. albicans binding forces were significantly lower for mannan-defective mutant C. albicans in comparison to the wild type (11). Binding forces of GtfB to *O*- or *N*-mannan mutant strains ranged from ∼0.2 nN to ∼0.5 nN, which were several-fold less compared with wild type (1 to 2 nN) (11). Thus, we first determined the binding forces of GtfB-C. albicans cell wall surface using single-molecule AFM to confirm whether disruption of GtfB-to-mannan binding is the driving mechanism for MDE antibiofilm activity. Encouragingly, our data matched the trend from previous results; the endoenzyme β-mannanase treatment of C. albicans reduced the binding forces of GtfB-to-C. albicans by ∼15-fold and increased GtfB binding failure by ∼2-fold (versus the vehicle control; Fig. 6D). These binding force values were comparable to the values for *N*-mannan mutant strain *och1*ΔΔ determined previously (11). Likewise, treatment of C. albicans with either of the exoenzymes α- or β-mannosidase led to significant reductions in the GtfB binding forces (∼5-fold) versus the vehicle control. These binding force values were similar to those for *O*-mannan mutant strains *pmt1*ΔΔ or *pmt4*Δ, demonstrating the efficacy of MDE to target the mannan structure on the fungal surface.

The observed differences in MDE efficiencies against cross-kingdom biofilm and GtfB binding are possibly due to the cleavage characteristics of the MDEs. Since β-mannanase as an endoenzyme could randomly hydrolyze internal/intramolecular mannosidic linkages, it might induce the detachment of bulky mannans from C. albicans. In contrast, the exoenzymes α-mannosidase and β-mannosidase could only degrade terminal linkages to liberate residues gradually, resulting in reduced removal of mannans (versus β-mannanase). We then hypothesized that such effects at the single-cell level would also affect the mechanical properties of the biofilm as a whole. Using a fluid shear-inducing device (11, 23), we observed a significant reduction in mechanical strength of β-mannanase treated biofilms, with increased surface detachment under mild shear stress (Fig. 4). This indicates that MDE strategy can also compromise the biofilm bulk stability, facilitating biofilm removal from the apatitic surface. Collectively, the data clearly show that disruption of the GtfB-mannan interactive ligand-receptor domain effectively impairs the interkingdom adhesion mechanism while also affecting biofilm mechanical integrity.

Receptor-specific targeting of the surface of C. albicans would be critical for the successful intervention of GtfB-C. albicans interaction using MDEs. Although both fungal and mammalian cells glycosylate proteins via similar mechanisms, a key difference is that *N*-linked and *O*-linked glycans on fungal (but not in mammalian) proteins are predominately composed of mannose (29). Since MDEs exhibit high specificity to mannose, it is likely that MDEs preferably bind to and hydrolyze mannose on fungal cells. However, there are other glycoproteins in saliva, and future studies should investigate whether the efficacy of MDEs is affected by other potential competitive substrates in the oral cavity. Meanwhile, we also explored whether the MDE approach could work with clinical isolates of S. mutans from ECC plaque that may have distinctive phenotype and biological properties. Excitingly, β-mannanase treatment was equally effective against biofilms formed with S. mutans clinical isolates (PDM1 and PDM4) compared to the ones with S. mutans UA159 (Fig. S8). However, further studies are needed to investigate additional S. mutans clinical variants, as well as the effects on other surface proteins/structures associated with this cross-kingdom interaction (30).

10.1128/mBio.00651-21.8 FIG S8 Efficacy of β-mannanase against S. mutans*-*C. albicans biofilms formed with the reference strain (UA159) or clinical isolates (PDM1 or PDM4) of S. mutans. (A) the pH of biofilm supernatant, (B) dry weight per biofilm, CFU of (C) S. mutans, and (D) C. albicans per biofilm. At optimal enzyme unit, β-mannanase had a significant antibiofilm effect on S. mutans*-*C. albicans biofilms as measured at 18, 28, and 42 h. VC and M refer to vehicle control and β-mannanase treated, respectively. Statistics: *** represents *P < *0.001 for unpaired *t* tests against the vehicle control (*n* ≥ 3). Download FIG S8, DOCX file, 0.3 MB. Copyright © 2021 Kim et al. 2021 Kim et al. https://creativecommons.org/licenses/by/4.0/ This content is distributed under the terms of the Creative Commons Attribution 4.0 International license.

In addition, enzyme stability in the oral environment would be equally important for therapeutic activity. Notably, MDEs maintained their enzymatic activities under physiologically relevant conditions (in complex human saliva; Fig. 3 and Fig. S2). Our data also show that MDEs were relatively stable under a nonoptimal buffer solution (MES buffer; Fig. S1), while maintaining catalytic activity across pH variations during biofilm growth, suggesting that the enzymes stay active under various surrounding environments. Despite their enzymatic stability, MDEs did not interfere with the growth and viability of S. mutans and C. albicans (Fig. S6 and S7), which could avoid the development of antimicrobial resistance over time. Moreover, the lack of cytotoxicity of MDEs toward human gingival keratinocytes (Fig. 7), in addition to preventive effects against tooth enamel demineralization, augurs well for its targeting specificity and potential clinical applications as a therapeutic agent. It is worth noting that MDEs at 5-fold higher concentrations than optimal units did not cause cytotoxicity (Fig. S9), which mitigates concern for the potentially deleterious effects of MDEs accumulation in the oral cavity.

10.1128/mBio.00651-21.9 FIG S9 Toxicity assay of 5-fold of the optimal units of MDEs on human gingival keratinocytes. Normalized cell viability for HGKs after exposure to 5-fold of the optimal units of MDEs for 1 h and 24 h. No significant loss in HGK cell viability was observed for 5× MDE treatments. Negative control and positive control represent vehicle control and 3% H_2_O_2_ control, respectively. Statistics: one-way ANOVA with *P < *0.01 *post hoc*; *, *P < *0.05; **, *P < *0.01 against vehicle control using Dunnett’s method (*n* ≥ 3). Download FIG S9, DOCX file, 0.1 MB. Copyright © 2021 Kim et al. 2021 Kim et al. https://creativecommons.org/licenses/by/4.0/ This content is distributed under the terms of the Creative Commons Attribution 4.0 International license.

Since MDEs hydrolyze mannose from the C. albicans surface, it is possible that cleaved mannoproteins can be utilized for bacterial growth and/or metabolic activity as reported elsewhere (31–34). However, the estimated amount of released mannoproteins from C. albicans is extremely low (∼500-fold less) compared with the supplemented carbon source (i.e., 1% sucrose or glucose). To test this, we extracted mannoproteins from C. albicans by β-mannanase and utilized them to compare the growth of S. mutans and Streptococcus gordonii and respective pH changes. As expected, we did not observe significant growth of either microorganism with limited pH drop when cultured in saliva supplemented with extracted mannoproteins. In contrast, those cultured in saliva with 1% glucose showed exponential growth and significant reduction of pH over time (Fig. S10).

10.1128/mBio.00651-21.10 FIG S10 Growth of S. mutans and S. gordonii and pH changes over time. Bacteria cultured in saliva supplemented with glucose showed exponential growth of bacteria and logarithmic reduction of pH over time. Bacteria cultured in saliva only or saliva supplemented with extracted mannoproteins from C. albicans via β-mannanase treatment were devoid of major effects (*n* ≥ 3). Download FIG S10, DOCX file, 0.2 MB. Copyright © 2021 Kim et al. 2021 Kim et al. https://creativecommons.org/licenses/by/4.0/ This content is distributed under the terms of the Creative Commons Attribution 4.0 International license.

Target specificity and retention of antibiofilm agents, as well as their penetration behaviors into the biofilm, may determine the fate of the antibiofilm strategy (35). For example, enhanced retention of antibacterial agent-loaded nanoparticles resulted in a dramatic improvement in antibiofilm activity, compared with the non-loaded antibacterial agent (36–38). Thus, enhanced retention and penetration of MDEs may further improve the efficacy of this approach. Future studies will assess the possibility of using nanocarriers to improve MDEs delivery. Finally, a previous study has shown that phagosome maturation is enhanced for C. albicans *O*-mannosylation mutant (defective in cell wall mannans) due to exposure of β-glucan in the inner cell wall (39). This finding indicates that MDEs could mitigate cellular inflammation caused by fungal-mediated infections. *In vivo* studies may provide further insights into this additional therapeutic effect.

In summary, our data revealed that targeting and intervening in the interkingdom receptor-ligand binding interactions using MDEs may lead to a novel nonbiocidal and more precise therapeutic measure. The enzymes are stable in complex human saliva and enzymatically active within a biofilm environment, efficiently degrading mannans on the C. albicans cell wall and, in turn, significantly impairing its binding potential with GtfB. The targeted disruption of receptor-ligand at the cellular level inflicted changes at macroscale that affected biofilm biomass, population, mechanical stability, and acidity, culminating with marked reduction of human tooth enamel demineralization. These properties were achieved without microbiocidal effects or causing cytotoxicity to human cells, suggesting a potential application as a targeted approach for disrupting a pathogenic cross-kingdom biofilm associated with severe ECC, a costly and unresolved oral infectious disease.

## MATERIALS AND METHODS

### Strains and culture conditions.

Candida albicans SC5314, a well-characterized fungal strain, and Streptococcus mutans UA159, a proven virulent cariogenic dental pathogen and well-characterized EPS producer, were used for biofilm experiments. Microbial stocks were stored at −80°C in tryptic soy broth containing 50% glycerol before use. All strains were grown to mid-exponential phase (optical densities at 600 nm [OD_600_] of 0.8 [C. albicans] and 1.0 [S. mutans], respectively) in ultrafiltered (10 kDa molecular mass cutoff; Millipore, Billerica, MA, USA) yeast-tryptone extract broth containing 2.5% tryptone and 1.5% yeast extract (UFYTE; pH 5.5 and 7.0 for C. albicans and S. mutans, respectively) with 1% (wt/vol) glucose at 37°C and 5% CO_2_, as described previously (24, 40). Cells were harvested by centrifugation (6,000 × *g*, 10 min, 4°C).

### Mannan-degrading enzymes (MDEs).

Purified exo-α-mannosidase (EC 3.2.1.24) was purchased from Sigma (MO, USA). Purified exo-β-mannosidase (EC 3.2.1.25) was purchased from Megazyme (Bray, Ireland). Purified endo-β-mannanase (EC 3.2.1.78) was purchased from Megazyme (Bray, Ireland). A unit of α-mannosidase activity is defined as the amount of enzyme required to release 1 μmol of p-nitrophenol (pNP) per min from p-nitrophenyl-α-d-mannopyranoside (5 mM) in MES buffer (100 mM) and CaCl_2_ (2.5 mM) at pH 6.5 at 40°C. A unit of β-mannosidase activity is defined as the amount of enzyme required to release 1 μmol of pNP per min from p-nitrophenyl-β-d-mannopyranoside (0.8 mM) in sodium maleate buffer (100 mM) at pH 6.5 at 35°C, monitored at 400 nm. A unit of β-mannanase activity is defined as the amount of enzyme required to release 1 μmol of mannose reducing-sugar equivalents per minute from carob galactomannan in sodium phosphate buffer (100 mM), pH 7.0 at 40°C.

### Saliva collection.

The study protocol was reviewed and approved by the Institutional Review Board of the University of Pennsylvania (protocol number 818549). Written informed consent was obtained from all volunteers in this study. Saliva was collected from healthy donors who had not taken any medications for at least a month. The donors chewed unflavored paraffin wax and saliva was collected in a conical tube on ice. Saliva was collected in the morning without having breakfast. Collected saliva was centrifuged (5,500 × *g*, 4°C, 10 min), followed by filter sterilization (0.22 μm; S2GPU01RE ultra-low-binding protein filter; Millipore, Billerica, MA). Filtered saliva was then kept at 4°C until use.

### C. albicans cell wall degradation assay.

C. albicans were grown to mid-exponential phase (optical densities at 600 nm of 0.8) in UFYTE, pH 5.5, containing 1% (wt/vol) glucose. An aliquot (1 mL) of the cell suspension was centrifuged at 10,000 × *g* for 10 min at 4°C. The cell pellet was resuspended and washed in the same volume of 1× PBS buffer (Dulbecco’s phosphate-buffered saline, 1×, Corning Inc., Corning, NY, USA) with pH of 7.33. This procedure was repeated twice to remove any remaining sugar. After treatment with MDEs (MES buffer, pH 6.5, 37°C, 5 min), the supernatant was collected and the cell pellet was resuspended in the same volume of MES buffer. All the supernatants were pooled and three volumes of cold ethanol were added, and the resulting precipitate was collected and resuspended in water. These precipitates were polysaccharides released from the cell wall after enzymatic treatments. Mannans from pellets were isolated using a mild alkali extraction method with boiling for 60 min (41, 42). Harvested pellets were washed with 1× PBS and then resuspended in 2% (wt/vol) KOH. This suspension was boiled for 60 min to extract mannan. The amount of reducing sugars was determined by the Somogyi-Nelson colorimetric assay (43, 44).

### Estimation of GtfB binding potential.

An overnight culture of C. albicans was subcultured to an OD of 0.8. The subculture was centrifuged (5,500 × *g*, 4°C, 10 min) followed by a wash with 1× PBS to remove all the nutrient medium and resuspended in 3 mL of MES buffer (prewarmed at 37°C). The suspension was split into 0.5 mL aliquots and respective MDEs were added at optimal units for 5 min (incubated at 37°C). Samples were then spun down and washed with 1× PBS to remove all the enzymes. The pellets were resuspended in 0.4 mL of adsorption buffer and incubated with 25 μg/mL of GtfB for 30 min at 37°C. Samples were then spun down and washed with 1× PBS to remove all the GtfB. Next, the pellets were resuspended in 0.5 mL of sucrose substrate for 1 h at 37°C. Samples were then spun down and washed with 1× PBS. Next, the pellets and formed glucans were resuspended in 1 mL of 1 N NaOH. Lastly, glucans formed were estimated colorimetrically as detailed previously (21).

### *In vitro* biofilm model.

Biofilms were formed using our saliva-coated hydroxyapatite (sHA) model as described previously (21). For HA disc (surface area, 2.7 ± 0.2 cm^2^; Clarkson Chromatography Products, Inc., South Williamsport, PA) coating, saliva was mixed with adsorption buffer at 1:1 ratio and clarified by centrifugation followed by filter sterilization as described previously. The HA discs were vertically suspended in 24-well plates using a custom-made wire disc holder, mimicking the free smooth surfaces of the pellicle-coated teeth. C. albicans were pretreated with each MDE for 5 min before inoculation. Each disc was inoculated with approximately 2 × 10^6^ CFU of S. mutans/mL and 2 × 10^4^ CFU of C. albicans/mL in prepared filter-sterilized saliva supplemented with 1% (wt/vol) sucrose at 37°C under 5% CO_2_. The proportion of the microorganisms in the inoculum is similar to that found in plaque samples from children with ECC (3, 12).

As illustrated in Fig. S3 in the supplemental material, the discs were treated with MDEs 3 times (6, 18, and 28 h) during biofilm formation. For enzyme treatment, each disc with biofilm was transferred to the prewarmed MES buffer (37°C) containing each enzyme, incubated for 5 min, and then transferred back to the cultured medium (6 h) or fresh medium (18 h and 28 h). For the vehicle control, each disc with biofilm was transferred to the prewarmed MES buffer not containing MDE (37°C), incubated for 5 min, and then transferred back to the cultured medium (6 h) or fresh medium (18 h and 28 h). The culture medium was changed twice daily at 8 a.m. and 6 p.m. and the pH of the supernatant was determined using an Orion pH electrode attached to an Orion DUAL STAR pH meter (Thermo Fisher Scientific, Waltham, MA, USA) until the end of the experimental period (42 h). The biofilms were collected at 18 h, 28 h, and 42 h for imaging and biochemical analysis.

In parallel, biofilms were also formed with two *S. mutans* clinical isolates from plaque samples collected from ECC children, and the efficacy of MDEs treatment was evaluated to further determine the feasibility of the clinical application. The study protocol was reviewed and approved by the Institutional Review Board of the University of Pennsylvania (protocol number 824243). These S. mutans clinical isolates were identified using Mitis Salivarius agar plus bacitracin (MSB) agar plates (11). All the biofilm experiments were performed following the procedures described above.

### Microbiological and biochemical biofilm analysis.

Collected biofilms at each time point were subjected to standard microbiological and biochemical analysis. Briefly, the biofilms were removed and homogenized by sonication, and the number of viable cells (CFU/biofilm) was determined (45). In parallel, an aliquot of biofilm suspension was centrifuged (5,500 × *g*, 10 min, 4°C), and the pellet was washed twice with Milli-Q water, dried in an oven (105°C, 24 h), and weighed. Quantification of polysaccharides was performed using an established colorimetric (phenol-sulfuric acid method) assay detailed previously (21). Three independent biofilm experiments were performed for each of the assays in duplicate.

### Confocal microscopy analysis.

The biofilms formed under each condition were examined using confocal laser scanning microscopy (CLSM) combined with quantitative computational analysis. Briefly, S. mutans cells were stained with 2.5 μM SYTO 9 green-fluorescent nucleic acid stain (485/498 nm; Molecular Probes Inc., Eugene, OR, USA) and C. albicans cells were stained with Concanavalin A (ConA) lectin conjugated with tetramethylrhodamine at 40 μg/mL (555/580 nm; Molecular Probes, Inc.), while EPS glucans were labeled with 1 μM Alexa Fluor 647-dextran conjugate (647/668 nm; Molecular Probes Inc.), as detailed previously (21). The confocal images of biofilms were obtained using an upright single-photon confocal microscope (LSM800, Zeiss, Jena, Germany) with a 20× (numerical aperture, 1.0) water objective. Each component was illuminated sequentially to minimize cross-talk as follows: SYTO 9 (S. mutans) was excited using 488 nm and was collected by a 480/40 nm emission filter; ConA (C. albicans) was excited using 560 nm and was collected by a 560/40 nm emission filter; Alexa Fluor 647 (EPS) was excited using 640 nm and collected by a 670/40 nm emission filter. Biofilm images were taken at 18 h after seeding microorganisms on the sHA discs in filtered saliva supplemented with 1% (wt/vol) sucrose. Images were subject to the quantification of biofilm biomass and visualization. Briefly, image stacks for each channel obtained using a Zeiss LSM800 were converted to 8-bit ome.tiff files and the COMSTAT plugin of ImageJ was used to generate values for biovolume (μm^3^/μm^2^). Biovolumes of S. mutans, C. albicans, and EPS glucans were quantified using COMSTAT2 as detailed elsewhere (40, 46–48). Three independent biofilm experiments were performed for each of the assays in duplicate.

### Analysis of the mechanical stability of biofilms.

The mechanical stabilities of S. mutans*-*C. albicans biofilms with or without MDE treatment were compared using a custom-built device (11, 23). Biofilms formed on sHA were placed in the disc holder of the device (Fig. 4A) and then exposed to a constant shear stress of 0.18 N/m^2^ for 10 min. The 10 min of shearing was previously determined as the duration sufficient to reach a steady-state for biofilm removal (11, 23). The amount of remaining biofilm dry weight (biomass) before and after application of shear stress was determined. Also, biofilms after application of shear stress were visualized using confocal microscopy as detailed in the previous section.

### Analysis of enamel surface demineralization.

Human tooth enamel blocks (4 mm × 4 mm) were prepared and coated with sterile clarified whole saliva (sTE). Cultures of ∼2 × 10^6^ CFU/mL of S. mutans and ∼2 × 10^4^ CFU/mL of C. albicans were grown on sTE in saliva supplemented with 1% sucrose (wt/vol), as detailed elsewhere (24). Briefly, biofilms were formed on enamel blocks mounted vertically at 37°C in 5% CO_2_ for 114 h. Biofilms were treated with PBS or β-mannanase as described in the “*In vitro* biofilm model” section. Saliva medium containing 1% sucrose was replaced twice daily until the end of the experiments. Then, biofilms were gently removed and the enamel slabs were collected for topography and surface roughness measurement. The surface topography and roughness of the enamel surface were analyzed by a nondestructive confocal contrasting method using Zeiss LSM 800 with a C Epiplan-Apochromat 50× (numerical aperture, 0.95) nonimmersion objective (24, 25). The images were processed using ConfoMap (Zeiss) to create 3D topography rendering and measure the surface properties in 3D. To quantify the surface demineralization, arithmetical mean height (*S_a_*) was measured using ISO 25178 (26). At least 3 independent experiments were performed for the assay.

### Atomic force microscopy and analysis.

Glass slides were coated with poly-l-lysine solution (0.1%; Sigma-Aldrich, St. Louis, MO, USA) by overnight incubation. C. albicans cells were immobilized on poly-l-lysine-coated glass slides (19) for 1 h at room temperature. Loosely adhered cells were removed by gentle washing with water and the slide was kept hydrated prior to AFM analysis. GtfB was prepared and purified via hydroxyapatite column chromatography, as detailed previously (17). AFM tips (TR400PSA, Olympus, Tokyo, Japan) were functionalized with 25 μg/mL of GtfB for 1 h at room temperature. Slides with immobilized C. albicans were incubated with optimal units of MDEs in MES buffer for 5 min at room temperature. Force measurements were then conducted under phosphate-buffered saline (HyClone Laboratories Inc., Logan, UT, USA) using an MFP-3D AFM (Asylum Research, Santa Barbara, CA, USA) as detailed elsewhere (19). Ten × ten adhesion force maps were obtained for 12 distinct cells from 3 distinct culture preparations. Force-distance curves were analyzed using AtomicJ (49).

### Microbicidal activity of MDEs on S. mutans and C. albicans.

To assess the effect of MDEs on the growth kinetics of the microbes, overnight cultures of S. mutans and C. albicans were subcultured until each reached optical densities (600 nm) of 1.0 and 0.8, respectively. Aliquots (1 mL) of the subcultures were spun down and treated with MDEs for 5 min at 37°C and pH 6.5. To remove MDEs, samples were subsequently spun down and the supernatants were discarded. Samples were then resuspended in 1 mL of fresh UFYTE medium and used to inoculate tubes with 9 mL of UFYTE medium. Growth curves were monitored for 6 h by measuring OD_600_ values every hour. To assess the effect of MDEs on CFU/mL of the microbes, a similar process was followed for MDE treatment. Samples were resuspended in 0.89% NaCl solution and viable cells (CFU/mL) were counted after 48 h.

### Cytotoxicity toward HGKs.

Immortalized human gingival keratinocytes (HGKs) were kindly provided by the laboratory of Dana T. Graves, School of Dental Medicine, University of Pennsylvania. HGK cells were seeded in 100 μL of KBM-2 medium (Lonza Group AG, Basel, Switzerland) with 0.15 μM CaCl_2_ (5,000 cells/well; 96-well plate format). The next day, the medium was discarded and optimal units or 5-fold of the optimal units of MDEs were added in serum-free KBM-2 for the treatment time (1 h and 24 h). After treatment, well volumes were replaced with fresh serum-free KBM-2 medium and left for a total of 24 h. The next day, 10 μl of 3-(4,5-dimethyl-2-thiazolyl)-2,5-diphenyl-2H-tetrazolium bromide (MTT) reagent (Sigma-Aldrich, St. Louis, MO, USA) was added to 90 μL of fresh serum-free KBM-2 medium. Samples were left for 5 h. Well volumes were then replaced with dimethyl sulfoxide (DMSO; Sigma-Aldrich, St. Louis, MO, USA). Absorbance values were read using a BioTek Elx800 (BioTek Instruments, Inc., Winooski, VT, USA). Percentage cell viability was calculated from the absorbance readings. Three independent experiments were conducted in triplicate.

### Effect of cleaved mannoproteins from C. albicans by β-mannanase on bacterial growth and pH changes.

A subculture of C. albicans was centrifuged (5,500 × *g*, 4°C, 10 min) followed by a wash with 1× PBS to remove all the nutrient medium and resuspended in 5 mL of filter-sterilized saliva (prewarmed at 37°C). β-mannanase at optimal unit was added to the suspension and incubated for 5 min (at 37°C). C. albicans were then spun down and supernatants were collected. Aliquots of ∼10^6^ cells of S. mutans or S. gordonii were incubated in saliva, saliva supplemented with 1% glucose, or saliva supplemented with cleaved mannoproteins from C. albicans. Optical densities and pH of bacterial cultures were recorded every 2 h.

### Statistical analysis.

Statistical analyses were carried out using GraphPad Prism 8 using one-way ANOVA (*post hoc* Dunnett’s method) and Student’s *t* tests where appropriate.
