# Supplementary material for: Intervening in Symbiotic Cross-Kingdom Biofilm Interactions: a Binding Mechanism-Based Nonmicrobicidal Approach
Source: mBio. 2021 May 18;12(3):e00651-21. doi: 10.1128/mBio.00651-21 (PMC8262967; doi:10.1128/mBio.00651-21)
Supplement: FIG S1 [file mbio.00651-21-sf001.docx]

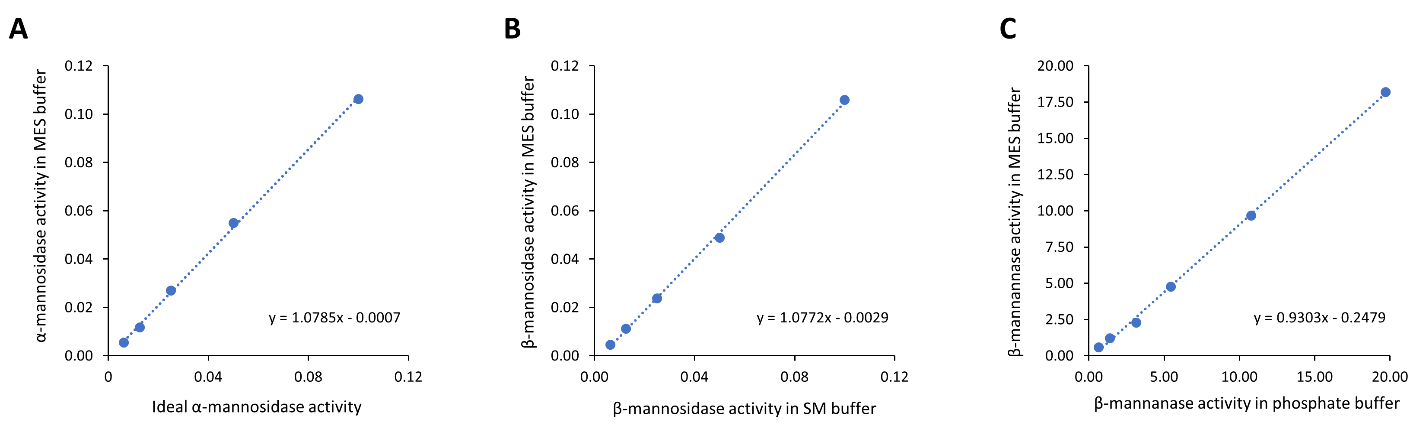


**Figure S1: Enzyme activity in MES versus recommended buffer.** Activities were compared for **(A)** *α*-mannosidase, **(B)** *β*-mannosidase, and **(C)** *β*-mannanase. All MDEs displayed similar activity in MES buffer when compared to the recommended buffer by the manufacturer (n≥3).
