# Supplementary material for: Intervening in Symbiotic Cross-Kingdom Biofilm Interactions: a Binding Mechanism-Based Nonmicrobicidal Approach
Source: mBio. 2021 May 18;12(3):e00651-21. doi: 10.1128/mBio.00651-21 (PMC8262967; doi:10.1128/mBio.00651-21)
Supplement: FIG S2 [file mbio.00651-21-sf002.docx]

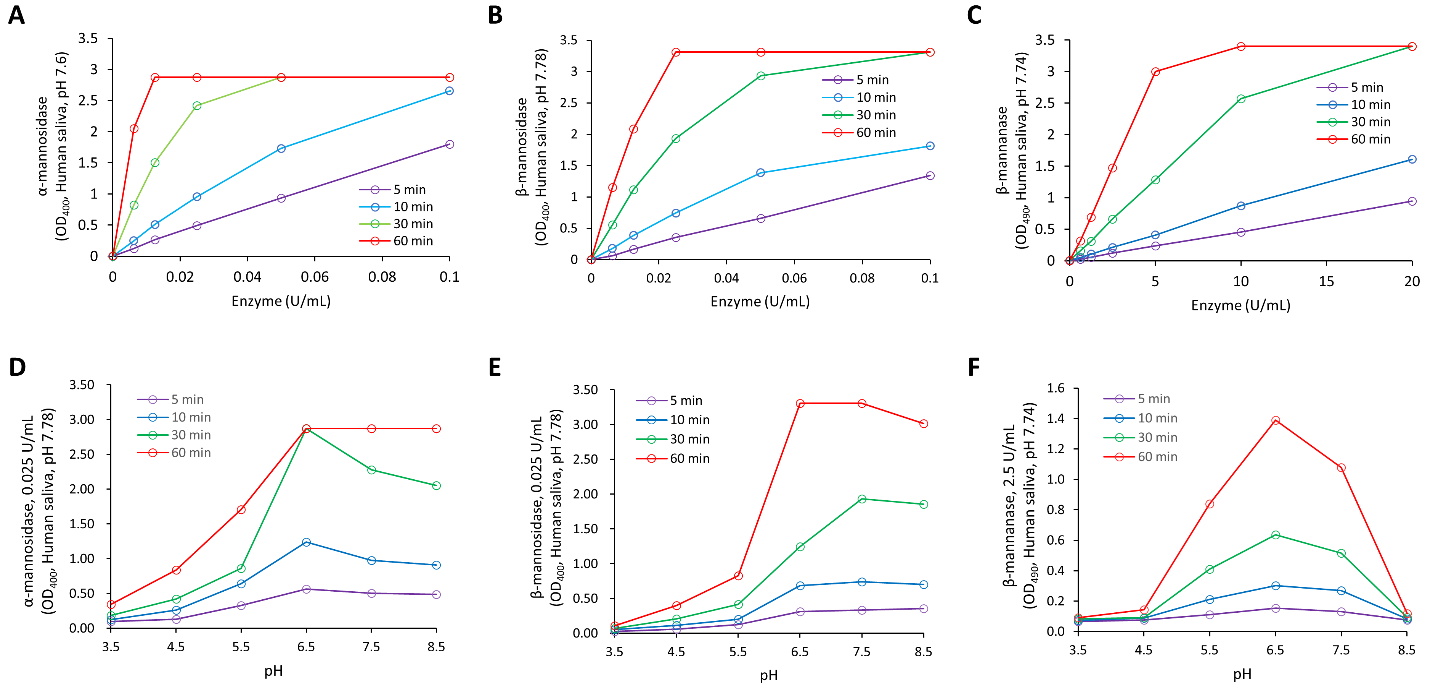


**Figure S2: Activity profiles for MDEs in saliva.** Activities were measured at different time points for **(A)** *α*-mannosidase, **(B)** *β*-mannosidase, and **(C)** *β*-mannanase. All MDEs had similar activity profiles for all time points. pH profiles were measured for **(D)** *α*-mannosidase, **(E)** *β*-mannosidase, and **(F)** *β*-mannanase. All the profiles in saliva were similar to the profiles in MES buffer (n≥3).
