# Supplementary material for: Intervening in Symbiotic Cross-Kingdom Biofilm Interactions: a Binding Mechanism-Based Nonmicrobicidal Approach
Source: mBio. 2021 May 18;12(3):e00651-21. doi: 10.1128/mBio.00651-21 (PMC8262967; doi:10.1128/mBio.00651-21)
Supplement: FIG S3 [file mbio.00651-21-sf003.docx]

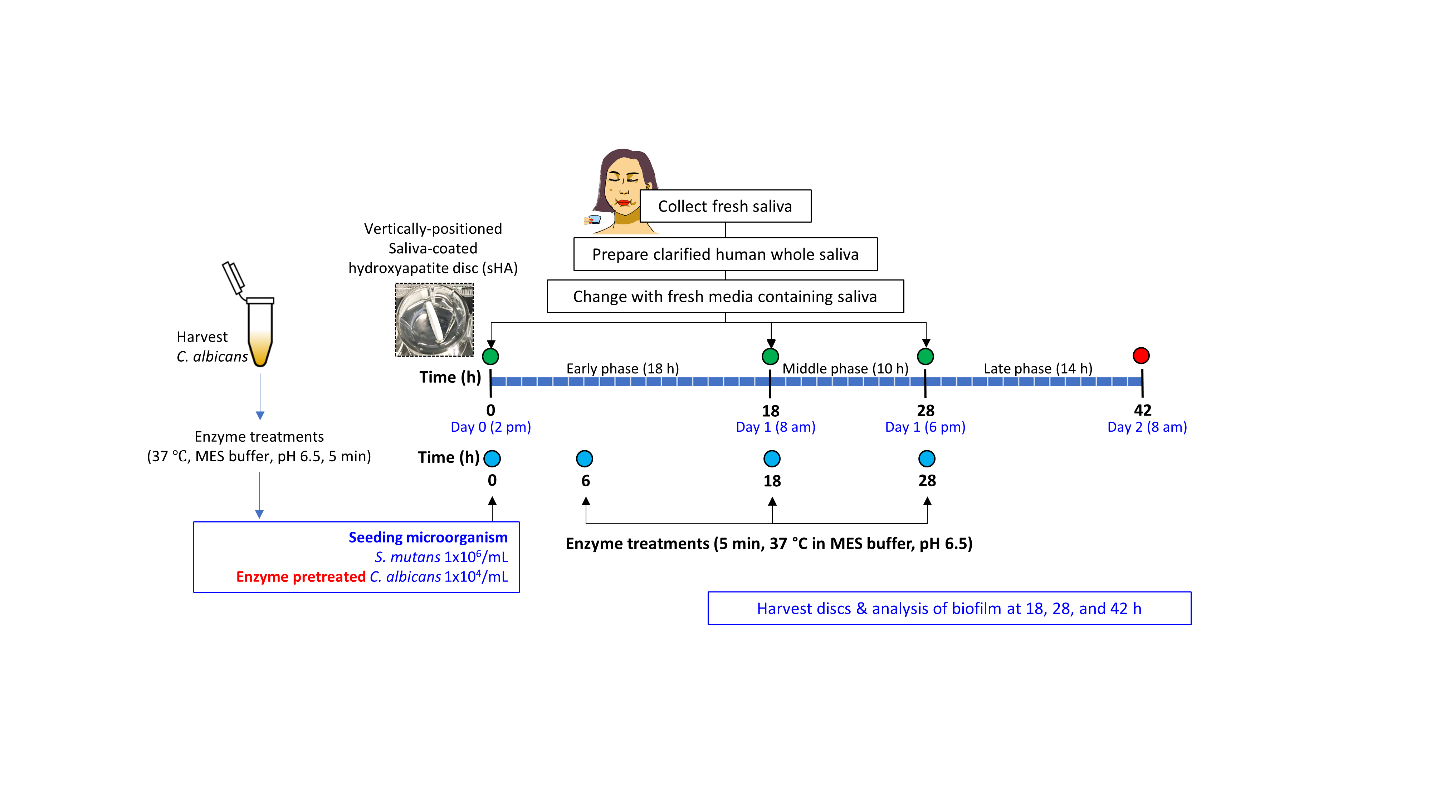


**Figure S3: Procedure to measure the antibiofilm activity of MDEs.** *C. albicans* were pre-treated with MDEs for 5 min before seeding. MDE treatments were then carried out at 6, 18 and 28 h.
