# Supplementary material for: Intervening in Symbiotic Cross-Kingdom Biofilm Interactions: a Binding Mechanism-Based Nonmicrobicidal Approach
Source: mBio. 2021 May 18;12(3):e00651-21. doi: 10.1128/mBio.00651-21 (PMC8262967; doi:10.1128/mBio.00651-21)
Supplement: FIG S4 [file mbio.00651-21-sf004.docx]

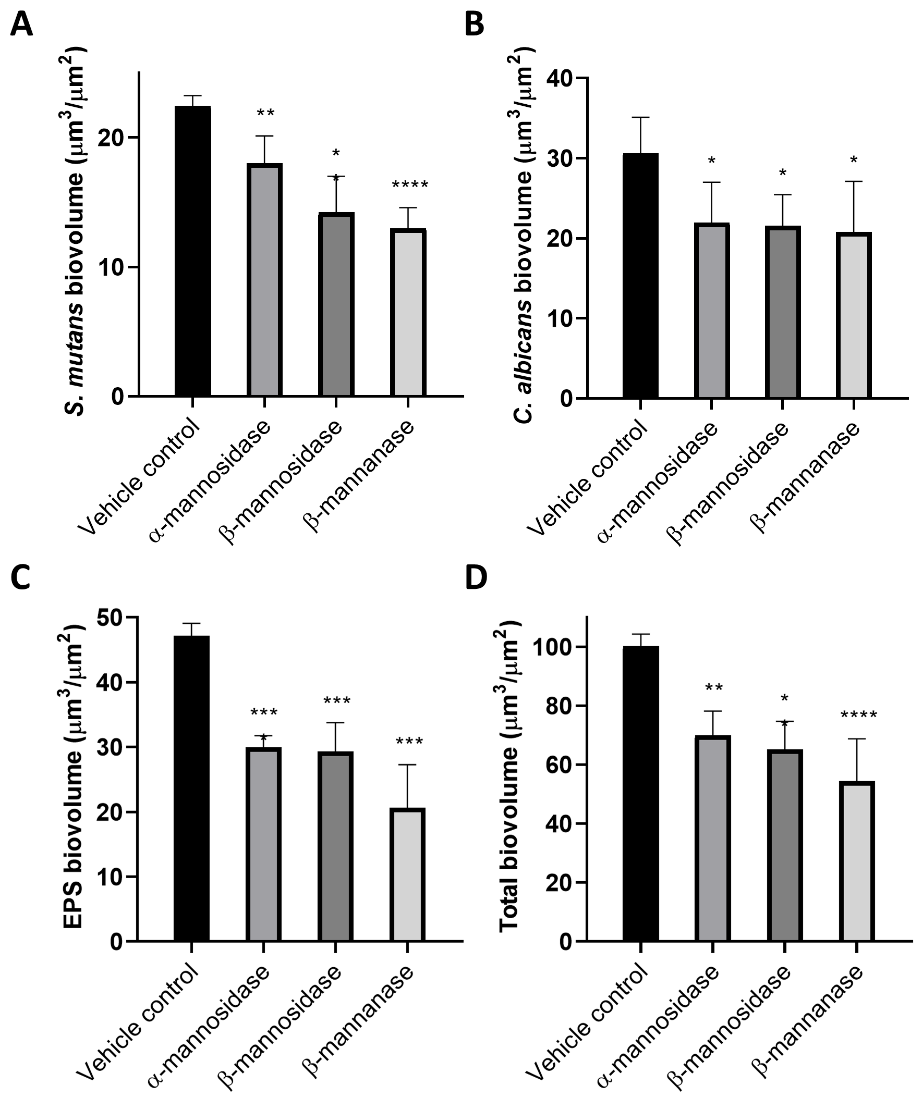


**Figure S4: Quantification of biovolumes for *S. mutans*, *C. albicans* and EPS with MDEs treatment.** Biovolumes (μm^3^/μm^2^) from confocal images for **(A)** *S. mutans*, **(B)** *C. albicans*, **(C)** EPS, and **(D)** total. All MDEs led to a reduction in biovolume of *S. mutans*, *C. albicans* and EPS. Statistics: one-way ANOVA with *P*<0.0001 *post hoc*; *, *P*<0.05; **, *P*<0.01; ***, *P*<0.001; **** *P*<0.0001 against vehicle control using Dunnett’s method (n≥3).
