# Supplementary material for: Intervening in Symbiotic Cross-Kingdom Biofilm Interactions: a Binding Mechanism-Based Nonmicrobicidal Approach
Source: mBio. 2021 May 18;12(3):e00651-21. doi: 10.1128/mBio.00651-21 (PMC8262967; doi:10.1128/mBio.00651-21)
Supplement: FIG S5 [file mbio.00651-21-sf005.docx]

**Figure S5: Efficacy of MDEs against *S. mutans-C. albicans* biofilms on human enamel slab.** **(A)** the pH of biofilm supernatant, **(B)** dry weight per biofilm, CFU of **(C)** *S. mutans* and *C. albicans* per biofilm. Statistics: ** represents *P*<0.01 for unpaired t-tests against the vehicle control (n≥3).
