# Supplementary material for: Intervening in Symbiotic Cross-Kingdom Biofilm Interactions: a Binding Mechanism-Based Nonmicrobicidal Approach
Source: mBio. 2021 May 18;12(3):e00651-21. doi: 10.1128/mBio.00651-21 (PMC8262967; doi:10.1128/mBio.00651-21)
Supplement: FIG S6 [file mbio.00651-21-sf006.docx]

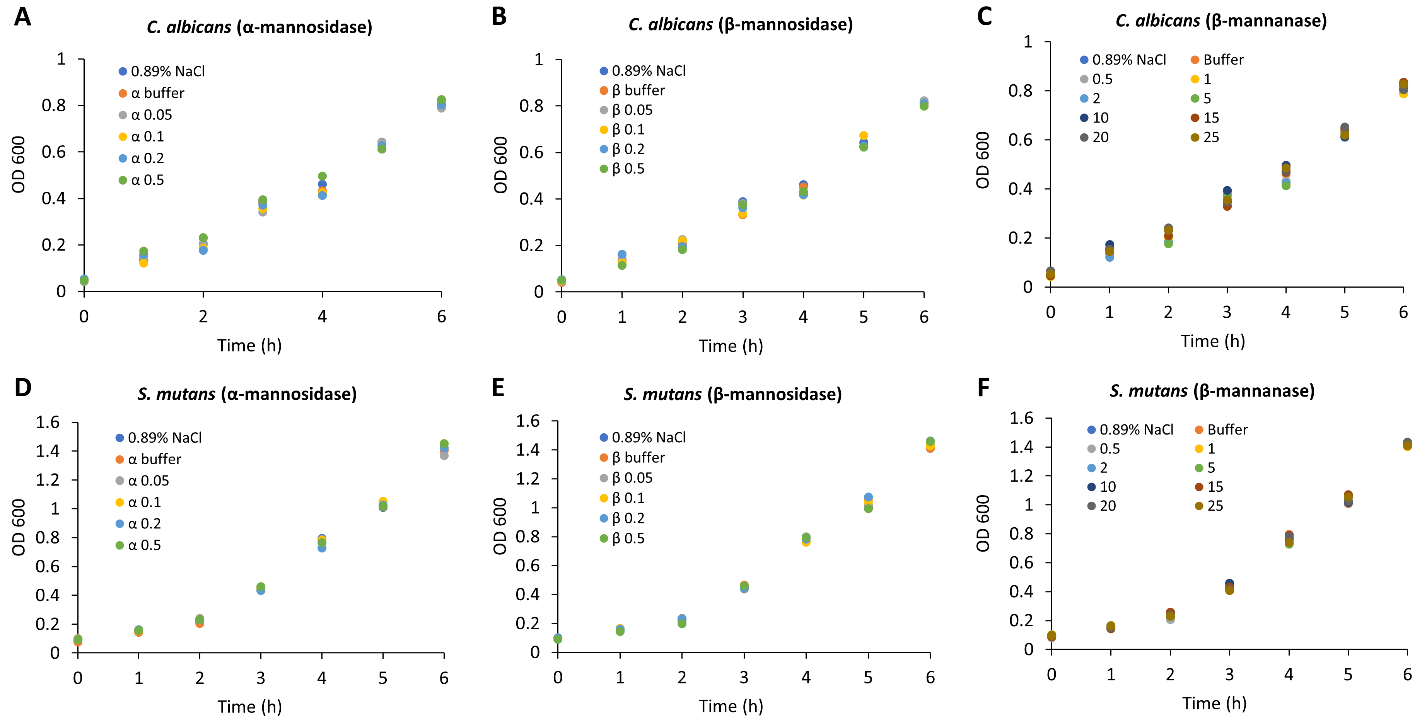


**Figure S6: Growth kinetics of *S. mutans* and *C. albicans* after treatment with MDEs.** Growth curves for *C. albicans* after treatment with **(A)** *α*-mannosidase, **(B)** *β*-mannosidase, and **(C)** *β*-mannanase. Growth curves for *S. mutans* after treatment with **(D)** *α*-mannosidase, **(E)** *β*-mannosidase, and **(F)** *β*-mannanase. All MDEs did not affect the growth curves of both microorganisms (n≥3).
