# Supplementary material for: Intervening in Symbiotic Cross-Kingdom Biofilm Interactions: a Binding Mechanism-Based Nonmicrobicidal Approach
Source: mBio. 2021 May 18;12(3):e00651-21. doi: 10.1128/mBio.00651-21 (PMC8262967; doi:10.1128/mBio.00651-21)
Supplement: FIG S7 [file mbio.00651-21-sf007.docx]

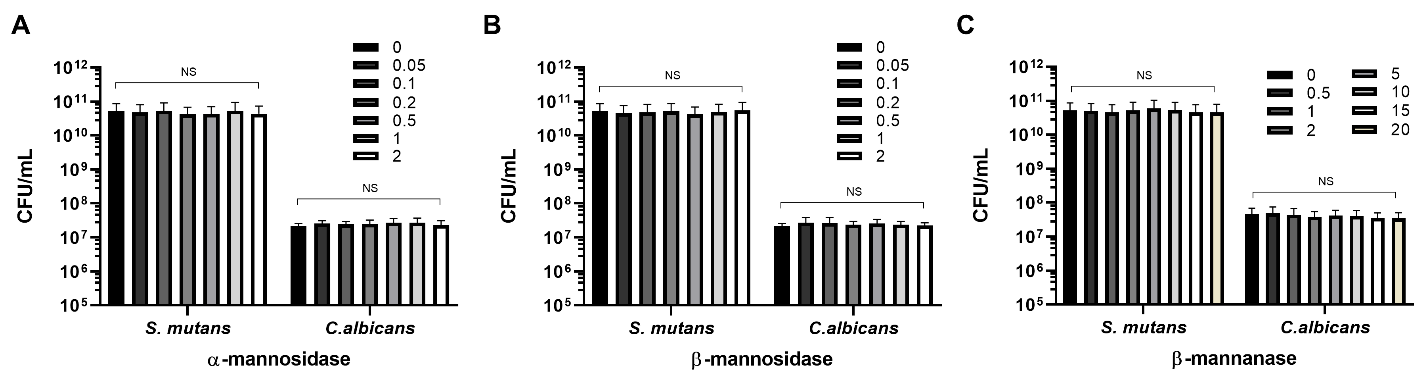


**Figure S7: Microbicidal activity of MDEs at different units.** CFU/mL for both *S. mutans* and *C. albicans* remained unchanged with the addition of **(A)** *α*-mannosidase, **(B)** *β*-mannosidase, and **(C)** *β*-mannanase. MDEs did not lead to a reduction in CFU/mL for both microbes (n≥3).
