# Supplementary material for: Intervening in Symbiotic Cross-Kingdom Biofilm Interactions: a Binding Mechanism-Based Nonmicrobicidal Approach
Source: mBio. 2021 May 18;12(3):e00651-21. doi: 10.1128/mBio.00651-21 (PMC8262967; doi:10.1128/mBio.00651-21)
Supplement: FIG S8 [file mbio.00651-21-sf008.docx]

**

**Figure S8: Efficacy of *β*-mannanase against *S. mutans-C. albicans* biofilms formed with reference (UA159) strain or clinical isolates (PDM1 or PDM4) of *S. mutans*.** **(A)** the pH of biofilm supernatant, **(B)** dry weight per biofilm, CFU of **(C)** *S. mutans*, and **(D)** *C. albicans* per biofilm. At optimal enzyme units, *β*-mannanase had a significant antibiofilm effect on *S. mutans-C. albicans* biofilms as measured at 18, 28, and 42h. VC and M refer to vehicle control and *β*-mannanase treated, respectively. Statistics: *** represents *P* < 0.001 for unpaired t-tests against the vehicle control (n≥3).
