# Supplementary material for: Intervening in Symbiotic Cross-Kingdom Biofilm Interactions: a Binding Mechanism-Based Nonmicrobicidal Approach
Source: mBio. 2021 May 18;12(3):e00651-21. doi: 10.1128/mBio.00651-21 (PMC8262967; doi:10.1128/mBio.00651-21)
Supplement: FIG S9 [file mbio.00651-21-sf009.docx]

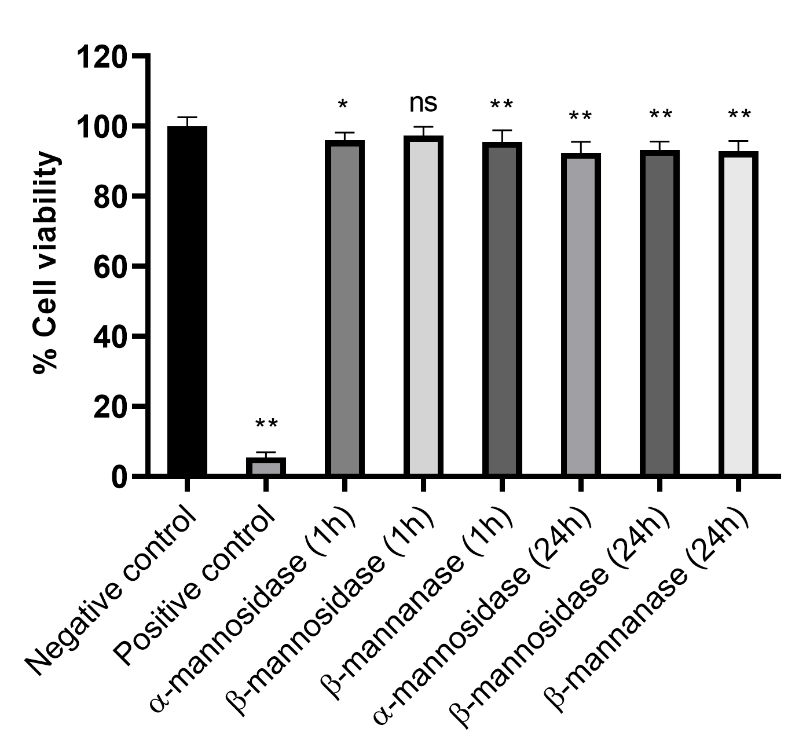


**Figure S9: Toxicity assay of 5-fold of the optimal units of MDEs on human gingival keratinocytes.** Normalized cell viability for HGKs after exposure to 5-fold of the optimal units of MDEs for 1 h and 24 h. No significant loss in HGK cell viability was observed for 5X MDE treatments. Negative control and positive control represent vehicle control and 3% H_2_O_2_ control, respectively. Statistics: one-way ANOVA with *P* < 0.01 *post hoc*; *, *P*<0.05 **, *P*<0.01 against vehicle control using Dunnett’s method (n≥3).
