# Supplementary material for: Intervening in Symbiotic Cross-Kingdom Biofilm Interactions: a Binding Mechanism-Based Nonmicrobicidal Approach
Source: mBio. 2021 May 18;12(3):e00651-21. doi: 10.1128/mBio.00651-21 (PMC8262967; doi:10.1128/mBio.00651-21)
Supplement: FIG S10 [file mbio.00651-21-sf010.docx]

**
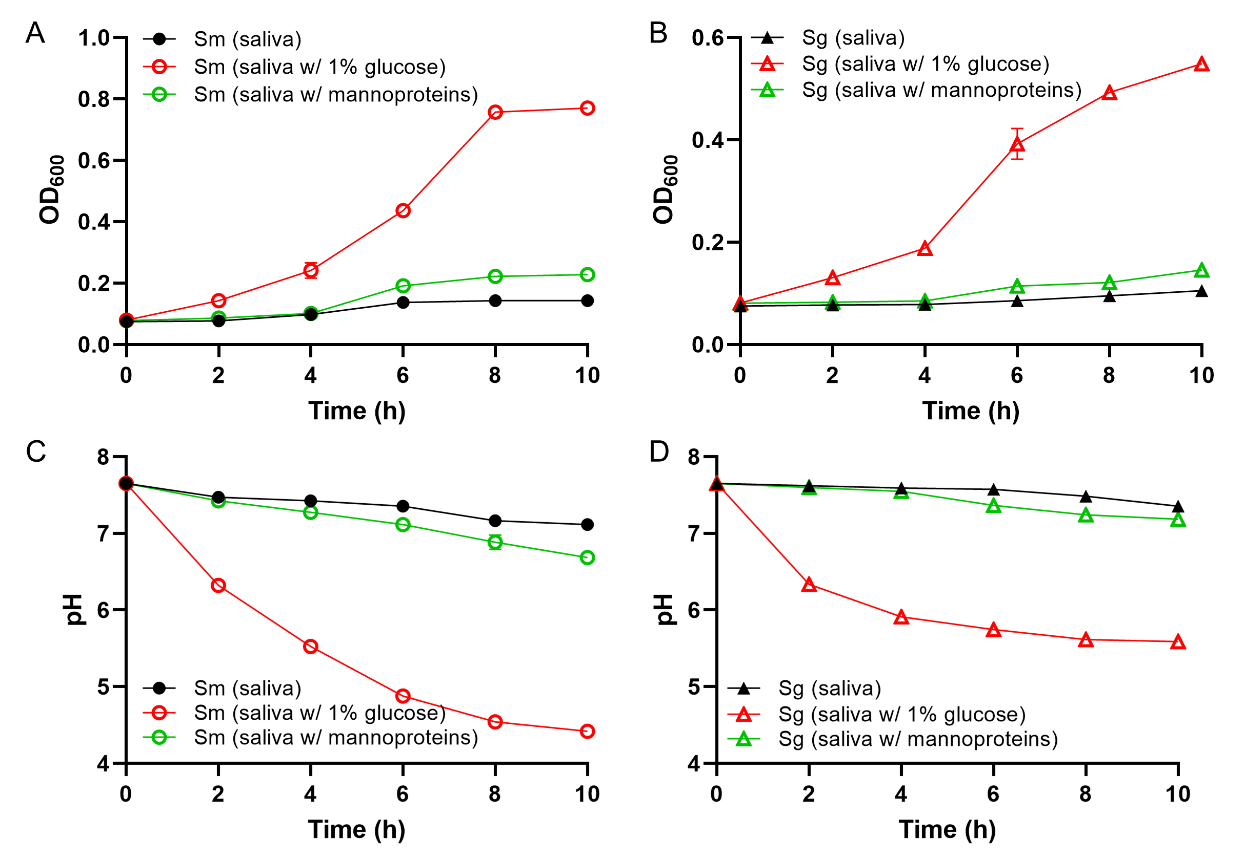
**

**Figure S10: Growth of *S. mutans* and *S. gordonii* and pH changes over time.** Bacteria cultured in saliva supplemented with glucose showed exponential growth of bacteria and logarithmic reduction of pH over time. Bacteria cultured in saliva only or saliva supplemented with extracted mannoproteins from *C. albicans* via *β*-mannanase treatment were devoid of major effects (n≥3).
